# Phosphate and acidosis cause fibre type‐specific changes to cellular and molecular contractile mechanics at 37°C in skeletal muscle from older adults

**DOI:** 10.1113/EP093983

**Published:** 2026-06-17

**Authors:** Brent A. Momb, Jane A. Kent, Stuart R. Chipkin, Mark S. Miller

**Affiliations:** ^1^ Department of Kinesiology University of Massachusetts Amherst Amherst Massachusetts USA

**Keywords:** ageing, fatigue, fibre, human, myosin heavy chain, temperature

## Abstract

Intracellular accumulation of hydrogen ions (H^+^) and inorganic phosphate (P_i_) has temperature‐dependent effects on single‐fibre contractile function between 10°C and 30°C. In vivo, human skeletal muscle temperatures range between 35‐39°C, and although contractile function is highly dependent on temperature, the effects of fatigue‐inducing [H^+^] and [P_i_] on contractile mechanics at 37°C are unknown. Using sinusoidal analysis, the independent and combined effects of these metabolites on cellular and molecular contractile function were determined at 37°C in slow‐contracting myosin heavy chain (MHC) I and fast‐contracting MHC IIA fibres from vastus lateralis muscle of 13 older adults (8 females), in four conditions: maximal calcium activation (‘control’; 5 mM P_i_, pH 7.0), high P_i_ (30 mM), low pH (6.2) and fatigue (30 mM P_i_ and pH 6.2). Specific tension (force/cross‐sectional area, mN/mm^2^) in both fibre types was reduced only in the fatigue condition (20%–26%). MHC I fibres had slower cross‐bridge kinetics, with fewer or less stiff strongly bound myosin–actin cross‐bridges in high P_i_, low pH and fatigue. The rate of myosin force production was slowest in low pH and fatigue conditions, whereas the myosin detachment rate was most altered by low pH alone. This indicates that during fatigue, high P_i_ moderates the slowing of cross‐bridge detachment owing to low pH. In contrast, fatigued MHC IIA fibres had faster cross‐bridge kinetics with increased myofilament viscosity. These findings quantify fibre type‐specific mechanical and kinetic mechanisms of fatigue in human skeletal muscle at 37°C, thus advancing our understanding of metabolite‐based muscle fatigue in vivo.

## Abstract (highlights)

- **What is the central question of this study?**
  How does the accumulation of hydrogen ions (H^+^) and/or inorganic phosphate (P_i_) affect skeletal muscle cellular and molecular contractile mechanics in different fibre types at a physiological temperature of 37°C?
- **What is the main finding and its significance?**
  Cross‐bridge kinetics, in general, decrease in slow‐contracting fibres and increase in fast‐contracting fibres in response to the accumulation of H^+^ and/or P_i_, which provides new insights into the mechanisms of fatigue in human skeletal muscle at a physiological temperature.

## 1 INTRODUCTION

Skeletal muscle fatigue is the reduced ability of a muscle to generate torque or power after contractile activity and is multifaceted, including impaired calcium (Ca^2+^) release and decreased neuronal activation, with the intracellular accumulation of inorganic phosphate (P_i_) and acidosis (high H^+^ or reduced pH) playing a primary role (Debold & Westerblad, 2024). Acute contractile failure in working human skeletal muscle can occur owing to the aforementioned increased P_i_ and reduced pH (Broxterman et al., 2017; Cady et al., 1989; Fitzgerald et al., 2023; Kent‐Braun, 1999; Weiner et al., 1990), which directly alter muscle contractile mechanics at the cellular and molecular levels (Foster et al., 2025; Sundberg et al., 2018, 2025) and thus, result in a loss of force‐generating capacity. Although both P_i_ and pH have temperature‐dependent effects on contractile function, the impact of these metabolites in single fibres has been studied primarily between 10°C and 30°C in animal models (Cooke et al., 1988; Coupland et al., 2001; Debold et al., 2004; Karatzaferi et al., 2008; Knuth et al., 2006), despite in vivo human skeletal muscle temperature ranging between 35‐39°C (Bergh & Ekblom, 1979; Flouris et al., 2015; Kenny et al., 2003). Furthermore, the response of different myosin heavy chain (MHC) isoforms to these metabolites has been of great interest in the field of muscle physiology, because the proportion of slow‐ or fast‐contracting fibres has been linked to the development of whole‐muscle contractile failure (Thorstensson & Karlsson, 1976). Despite this, potential fibre type‐specific responses to increased P_i_ and reduced pH have not been studied extensively. Given that the accumulation of P_i_ and reduced pH play a large role in muscle fatigue (Broxterman et al., 2017; Cooke, 2007; Kent‐Braun et al., 1993, 2002; Vandenboom, 2017), have temperature‐sensitive effects on contractile function (Nelson et al., 2014; Sundberg et al., 2018) and might cause different responses depending on fibre type (Mizuno et al., 1994a, 1994b), an understanding of how myosin and actin function at 37°C is crucial to understanding muscle fatigue.

The combined effects of high P_i_ and low pH on contractile function has been documented in single fibres from animals (Cooke et al., 1988; Nelson et al., 2014; Nocella et al., 2011; Potma et al., 1995) and humans (Foster et al., 2025; Sundberg et al., 2018, 2025), with studies indicating that their effect on force or contractile velocity generally diminishes as temperature is increased towards a physiological range from 15°C to 30°C (Nelson et al., 2014; Sundberg et al., 2018). Lower force in single fibres during fatigue induced by high P_i_ and low pH has been thought to be attributable to reduced force per cross‐bridge (Foster et al., 2025; Nelson et al., 2014; Nocella et al., 2011) and/or reductions in the number of cross‐bridges (Foster et al., 2025; Nocella et al., 2011) and might be fibre type specific (Foster et al., 2025). Although not all studies agree on the underlying mechanism, slowed contractile velocity is generally thought to be due to slowed cross‐bridge kinetics (Foster et al., 2025; Nelson et al., 2014; Sundberg et al., 2018). Until recently, only one research group had performed experiments on single fibres from humans at 30°C (Sundberg et al., 2018), and solely in slow‐contracting MHC I fibres, which might respond to metabolites in a different way from MHC IIA (Foster et al., 2025; Potma et al., 1995). Thus, a lack of information exists regarding how the combination of P_i_ and pH impact cellular‐ and molecular‐level mechanics in MHC I and MHC IIA fibres at in vivo temperatures.

Although P_i_ and pH generally do not change independently in vivo, the contractile response to each metabolite individually might provide insight into their combined behaviour by understanding which steps in the cross‐bridge cycle are altered. The effects of P_i_ or pH alone on contractile function have been examined extensively in single fibres from animals with a variety of techniques (Cooke & Pate, 1985; Debold et al., 2004; Karatzaferi et al., 2008; Knuth et al., 2006; Metzger & Moss, 1987, 1990; Wahr et al., 1997; Wang & Kawai, 2001; Westerblad et al., 1997; Widrick, 2002; Zhao & Kawai, 1994). However, limited data exist on alterting these metabolites alone in humans, because only one study has examined the effect of P_i_ alone and only in a small concentration range (0–4 mM P_i_; Sundberg et al., 2025), even though P_i_ has been shown to increase to 30 mM or more in vivo (Broxterman et al., 2017; Cady et al., 1989; Fitzgerald et al., 2023; Kent‐Braun, 1999; Weiner et al., 1990). Given that P_i_ and pH directly alter the rates of myosin attachment and detachment while knowing that the effects of each are temperature dependent (Debold et al., 2008; Foster et al., 2025; Marang et al., 2025; Wang & Kawai, 2001; Woodward & Debold, 2018; Zhao & Kawai, 1994), studying these metabolites at 37°C in humans will provide insight into their respective roles in muscle fatigue in conditions that mimic those in vivo.

Obtaining data in humans is important, because humans and animals have different skeletal muscle contractile properties (Pellegrino et al., 2003), including myosin–actin cross‐bridge kinetics, which tend to be slower as species increase in size (Nyitrai et al., 2006). Additionally, P_i_ (Coupland et al., 2001; Debold et al., 2004) and pH (Pate et al., 1995; Westerblad et al., 1997; Wiseman et al., 1996) have temperature‐dependent effects, such that as temperature increases their independent effects on single‐fibre force production and contractile velocity are diminished. Furthermore, rates of myosin attachment and detachment are highly temperature sensitive (Wang & Kawai, 2001; Zhao & Kawai, 1994) and directly impacted by the accumulation of P_i_ or H^+^ (Debold et al., 2008; Foster et al., 2025; Marang et al., 2025; Wang & Kawai, 2001; Woodward & Debold, 2018; Zhao & Kawai, 1994). Thus, studying these metabolites at 37°C in humans will provide insight into their respective roles in muscle fatigue in conditions that mimic those in vivo.

To date, the study of human skeletal muscle tissue at physiological temperatures has been limited to some extent owing to deterioration of MHC IIA fibres above 15°C–25°C and MHC I fibres at >30°C (Sundberg et al., 2018). To achieve a higher testing temperature of 37°C and maintain fibre stability in human single fibres, we chemically fix the ends of single fibres with a glutaraldehyde solution to cross‐link proteins (Miller et al., 2010). To quantify cross‐bridge mechanics and kinetics, we use small‐amplitude (0.05% of fibre length) sinusoidal perturbations of single fibres to reduce damage to fibres. Fibre damage can occur with other techniques that require large length changes (Sundberg et al., 2018), such as force–velocity curves or measurements of unloaded shortening velocity. Importantly, the sinusoidal analysis method permits the assessment of myofilament protein function in its native, three‐dimensional configuration at the level of the myosin–actin cross‐bridge. In calcium‐activated conditions, sinusoidal length perturbations, applied below the unitary myosin step size and across a range of frequencies (Brenner, 2006; Kawai, 2003), facilitate the measurement of muscle mechanical properties relevant to specific steps in the cross‐bridge cycle (Kawai et al., 1993; Mulieri et al., 2002; Palmer et al., 2007; Zhao & Kawai, 1993). The application of sinusoidal analysis at 37°C with fixation of single fibres in human skeletal muscle thus allows for a mechanistic understanding of the impact of elevated P_i_ and reduced pH on muscle contractile function in more physiologically relevant conditions.

The purpose of this study was to quantify the independent and combined effects of elevated P_i_ and reduced pH on cellular (single‐fibre) and molecular (myosin–actin cross‐bridge kinetics and mechanics) contractile function at 37°C in single human skeletal muscle fibres from healthy older adults. Slow‐contracting MHC I and fast‐contracting MHC IIA fibres from the vastus lateralis muscle were examined in four conditions of maximal calcium activation: control (5 mM P_i_, pH 7.0), high P_i_ (30 mM), low pH (6.2) and fatigue (30 mM P_i_ and pH 6.2). Control phosphate (P_i_) concentration was 5 mM to align with resting [P_i_] in healthy human gastrocnemius (5 mM) and quadriceps (4.5 mM) muscles (Kemp et al., 2007; Pathare et al., 2005). Fatigue values for P_i_ and pH were chosen because P_i_ has been shown to increase to 30 mM or more and pH to decline to as low as 6.2 in vivo during fatiguing contraction protocols in various human skeletal muscles expressing a range of MHC isoforms (Broxterman et al., 2017; Cady et al., 1989; Fitzgerald et al., 2023; Kent‐Braun, 1999; Weiner et al., 1990). Skeletal muscle biopsies were performed in healthy older participants (65–80 years of age), with habitual physical activity measured by accelerometry.

## 2 MATERIALS AND METHODS

### 2.1 Ethical approval

Written, informed consent was obtained from each volunteer before their participation. The protocol was approved by the Institutional Review Board at the University of Massachusetts Amherst (Institutional Review Board protocols 218 and 1758) and was performed following the *Declaration of Helsinki*, except for registration in a database.

### 2.2 Participants

Thirteen healthy older adults (five males and eight females) completed this study. All volunteers were aged 65–80 years, generally healthy by self‐report, ambulatory without the use of walking aids, living independently in the community, and sedentary to somewhat active (no more than two 30 min light‐ to moderate‐volitional exercise sessions per week by self‐report), as verified by accelerometry. Individuals were excluded if they had a history of major neurological, neuromuscular or orthopaedic conditions, cardiovascular disease or other metabolic diseases that could impact neuromuscular function, uncontrolled hypertension (blood pressure > 140/90 mmHg), smoking in the past year, moderate to severe lower‐extremity arthritis, pain, muscle cramps, joint stiffness, light‐headedness or other symptoms upon exertion, the use of β‐blockers, sedatives, tranquilizers or other medication that could impair physical function, body mass index > 35 kg/m^2^ (because increased fat mass might alter single muscle fibre performance) or body mass index < 18 kg/m^2^. Any persons taking anticoagulant medication or with known coagulopathies (owing to increased bleeding risk from the biopsy procedure), participants with a contraindication for magnetic resonance testing, including a pacemaker or other implant, males and females undergoing hormone replacement therapy (because this treatment might circumvent normal age‐related declines in sex hormone levels; if taken, hormone therapy must have been >5 years ago), unintentional weight loss of >2.5 kg during the last 3 months, currently participating or having participated in a weight‐loss or exercise training programme in the last year were also reasons for exclusion. All participants obtained clearance from their health‐care provider to participate. Eligibility was determined during a screening visit, followed by collection of informed consent and anthropometric data.

### 2.3 Accelerometry

To account for the potential confounder of physical activity on skeletal muscle function, participants were asked to complete a physical activity log and wear an accelerometer (ActiGraph GT3X‐BT) on their dominant hip for 7 days. Data were collected at 60 Hz in 1 s epochs. ActiLife software (ActiGraph, Pensacola, FL, USA) was used to calculate moderate‐to‐vigorous physical activity using the Troiano cut points (Troiano et al., 2008) from a minimum of 4 days, including 1 day at the weekend. All days included ≥10 h of wear time.

### 2.4 Experimental solutions

All solutions used for biopsy processing and single‐fibre experiments were calculated using the equations and stability constants according to Godt and Lindley (1982). Characteristics of the solutions used for dissection, skinning, storage and single‐fibre experiments are given in Table 1.

**TABLE 1:** Characteristics of solutions used for skeletal muscle fibres.

Solution	Constituent										
	BES (mM)	EGTA (mM)	CP (mM)	CPK (mL/mg)	DTT (mM)	Free Mg^2+^ (mM)	ATP (mM)	P_i_ (mM)	pCa	pH	Ionic strength (mequiv)
Dissecting	20	5	0	0	1	1	5	0.25	8.0	7.0	175
Relaxing	20	5	15	300	1	1	5	5	8.0	7.0	175
Pre‐activating	20	0.5	15	300	1	1	5	5	8.0	7.0	175
Activating control	20	5	15	300	1	1	5	5	4.5	7.0	175
Act. high P_i_ (30 mM)	20	5	15	300	1	1	5	30	4.5	7.0	175
Act. low pH (6.2)	20	5	15	300	1	1	5	5	4.5	6.2	175
Activating fatigue	20	5	15	300	1	1	5	30	4.5	6.2	175

	MgCl_2_ (mM)	EGTA (mM)	Potassium propionate (mM)	Imidazole (mM)	Na_2_H_2_ATP (mM)	Sodium azide (mM)	pCa	pH	Protease inhibitor
Storage	2.5	5	170	10	2.5	0	8.0	7.0	complete Mini
Skinning	2.5	5	170	10	2.5	1	8.0	7.0	–

*Note*: Ionic strength of 175 mEq was obtained using sodium methane sulphate.

Abbreviations: Act., activating; BES, *N*,*N‐bis*(2‐hydroxyethyl)‐2‐aminoethanesulfonic acid; cOmplete Mini, tablets from Roche; CP, creatine phosphate; CPK, creatine phosphokinase; DTT, dithiothreitol; pCa, −log10([Ca^2+^]).

### 2.5 Skeletal muscle tissue biopsy and processing

Volunteers fasted for ≥10 h prior to the muscle biopsy. Muscle tissue from one or two legs was obtained via percutaneous biopsy of the vastus lateralis under lignocaine anaesthesia. Skeletal muscle tissue was placed immediately into cold (4°C) dissecting solution. Muscle bundles were placed in skinning solution for 24 h at 4°C and were then placed in storage solutions with increasing concentrations of glycerol (10% v/v glycerol for 2 h, then 25% v/v glycerol for 2 h) until reaching the final storage solution (50% v/v glycerol) and incubated at 4°C for 18–20 h. Thereafter, bundles were stored at −20°C until isolation of single fibres for mechanical measurements, which occurred within 4 weeks of the biopsy.

### 2.6 Preparation of single fibres for mechanical analysis

Preparation of single fibres has been described previously (Miller et al., 2010). Briefly, muscle bundles were incubated in dissection solution containing 1% Triton X‐100 (v/v) for 30 min at 4°C. Segments (∼2.0–2.5 mm) of single fibres were isolated from muscle bundles manually, with aluminum T‐clips placed at both ends of the fibre. The fibre was again incubated in dissecting solution containing 1% Triton X‐100 (v/v) for 30 min at 4°C to ensure removal of the sarcolemma and sarcoplasmic reticulum. The fibre was then mounted onto hooks in dissecting solution at room temperature on the cross‐sectional area (CSA)/fixation rig. Top and side diameter measurements were made at three positions along the length of the middle 1–2 mm of the fibre using a filar eyepiece micrometer (Lasico, Los Angeles, CA, USA) and a right‐angled, mirrored prism to obtain the side‐to‐top width ratio. Fibres were then fixed at two points ∼1–2 mm apart with glutaraldehyde, at the end of each T‐clip. New T‐clips were placed on the fixed regions dyed with Bromophenol Blue. The fibre was then transferred to the sinusoidal analysis rig for mechanical testing.

### 2.7 Experimental protocol

Each fibre was tested for maximal specific tension (mN/mm^2^) at 37°C in each of the experimental conditions in a random order with minimal time (< 5 s) between each trial: Control (P_i_ = 5 mM, pH = 7.0), high P_i_ (P_i_ = 30 mM, pH = 7.0), low pH (P_i_ = 5 mM, pH 6.2) and fatigue (P_i_ = 30 mM, pH = 6.2). Myofilament mechanical properties, including myosin–actin cross‐bridge kinetics, were measured using small‐amplitude sinusoidal analysis in the same randomized order as the initial measurements for maximal specific tension. Sinusoidal analysis experiments took ∼40 s to complete at 37°C.

### 2.8 Single‐fibre mechanical analysis

Mechanical analysis of single muscle fibres was adapted from a previous study (Miller et al., 2010). The T‐clipped ends of the fibre were attached to a piezoelectric motor (Physik Instrumente, Auburn, MA, USA) and a strain gauge (SensorNor, Horten, Norway) on the sinusoidal analysis rig in relaxing solution at 15°C. The fibre was stretched manually until the sarcomere length was 2.65 µm (IonOptix, Milton, MA, USA), because this resembles in vivo sarcomere length in human skeletal muscle (Chen et al., 2016). A camera (Point Grey, FLIR Integrated Imaging Solutions, Inc., Richmond, BC, Canada) was used to image the fibre, and a computer program (ImageJ) performed a fast Fourier transformation of the visible light and dark sarcomere patterns to determine sarcomere length. Fibre length (*L*) was measured using a micrometer as the distance between the inside edges of the T‐clips on each end of the fibre. Fibre top width was measured in the middle of the fibre, and side width was estimated using the side‐to‐top width ratio obtained on the CSA/fixation rig. Fibre top and estimated side widths were used to calculate elliptical CSA.

**FIGURE 1:**
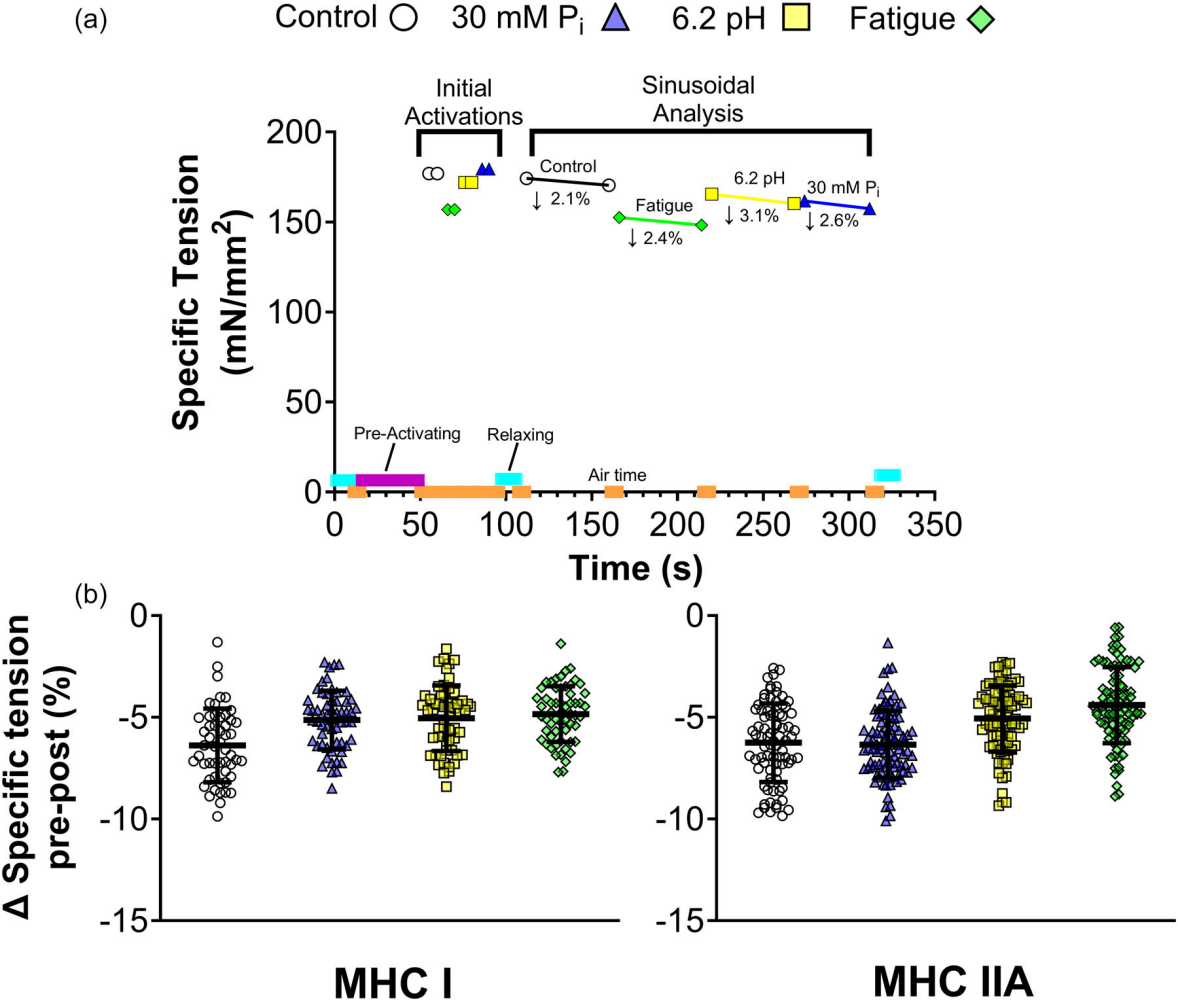
Experimental protocol for sinusoidal analysis and percentage reduction in single skeletal muscle fibre maximal Ca^2+^‐activated (pCa 4.5) specific tension from pre‐ to post‐sinusoidal analysis by MHC isoform for control, high P_i_, low pH and fatigue conditions at 37°C. (a) Data are presented for a single fibre undergoing the experimental protocol. Each single fibre underwent an initial activation at 15°C to ensure integrity (not pictured) prior to the experimental protocol at 37°C. Conditions were randomized for each single fibre. (b) Data are presented as the mean ± SD, with individual fibres as data points. The number of fibres (*n*) tested for each condition is as follows: MHC I, *n* = 58; and MHC IIA, *n* = 92. Abbreviations: MHC, myosin heavy chain; P_i_, inorganic phosphate.

Starting in relaxing solution at 15°C, the fibre was slackened completely, the force gauge zeroed, the fibre pulled back to its original position, allowed to equilibrate for 1 min and relaxed isometric force measured (e.g., setting the force baseline). The fibre was transferred to pre‐activating solution for 30 s, then to control activating solution, with force recorded at its plateau, because we have found that initial activation at 15°C helps to maintain fibre integrity over the course of the experiment. The fibre was returned to relaxing solution, given time to relax, and stretched as necessary to return sarcomere length to 2.65 µm. Starting again in relaxing solution, the temperature was increased to 37°C over the course of 1–2 min, and force baseline was set. At this point, the fibre was considered to be prepared for experimentation. The fibre was transferred from pre‐activating solution to one of the activating solutions, and specific tension was recorded at its plateau (Figure 1a). The fibre was immediately moved to the other activation conditions (control, high P_i_, low pH or fatigue) in a randomized order to obtain their specific tension values (Figure 1). After each specific tension was recorded, the fibre was transferred to each activating solution in the same randomized order, with small‐amplitude, sinusoidal length changes (0.05% of *L*) applied to the fibre at 42 frequencies (ranging from 3 to 200 Hz), with no relaxation between conditions but with relaxation at the end of the experiment. Each sinusoidal analysis run at 37°C took ∼40 s, allowing for maintenance of sarcomere integrity without compromising data collection. Specific tension was recorded prior to each sinusoidal analysis run and afterwards to ensure fibre stability. If specific tension declined by >10% of the initial value or sinusoidal analysis data could not be fitted adequately to the six‐parameter model, the fibre results were not included in further analysis. The change in specific tension from pre‐to‐post sinusoidal analysis for each condition is presented in Figure 1b.

Length and force were normalized to determine fibre strain (Δ*L*/*L*) and stress (force/CSA). Elastic (*E*_e_) and viscous (*E*_v_) moduli (N/mm^2^) were calculated from the stress transient by determining the magnitudes of the in‐phase and out‐of‐phase components (0° and 90° with respect to strain). The elastic and viscous moduli are the real and imaginary parts of the complex modulus, i.e., the ratio of the stress response to the strain. Myosin–actin cross‐bridge mechanics and kinetics and properties of the myofilaments were derived, as previously described (Miller et al., 2010; Momb et al., 2022), by fitting the complex modulus with the following six‐parameter equation:

(1) $$Y\left(\omega \right)=A{\left(i\omega /\alpha \right)}^{k}-Bi\omega /\left(2\pi b+i\omega \right)+Ci\omega /\left(2\pi c+i\omega \right)$$

where ω = 2π*f* in s^−1^, *A*, *B* and *C* are magnitudes expressed in N/mm^2^, 2π*b* and 2π*c* are characteristic rates expressed in s^−1^, *i* = −1^½^, α = 1/s, and *k* is a unitless exponent. This analysis yields three characteristic processes, A, B and C, which relate to various mechanical (*A*, *B*, *C* and *k*) and kinetic (2π*b* and 2π*c*) properties of the cross‐bridge cycle. Nyquist plots, or viscous versus elastic modulus, show the stress response of the fibre to strain over a range of frequencies, which can be fitted using the six‐parameter equation above. Process A (*A* and *k*) has no enzymatic dependence and, in Ca^2+^‐activated conditions, reflects the viscoelastic mechanical response of the structural elements of the muscle fibre (the myofilament lattice stiffness and attached myosin heads in series) (Mulieri et al., 2002; Palmer et al., 2004). The parameter *A* indicates the magnitude of the viscoelastic modulus, whereas *k* represents the degree to which these viscoelastic magnitudes are purely elastic (*k *= 0) or purely viscous (*k *= 1). The magnitudes (*B* and *C*) of processes B and C are proportional to the number of strongly bound myosin–actin interactions and/or the stiffness of the cross‐bridges (Kawai et al., 1993). The *C*/*B* ratio is the relationship between the magnitude of the work‐absorbing or positive viscous modulus, process C (*C*), and the work‐producing or negative viscous modulus, process B (*B*). The frequency portion of process B (2π*b*) is interpreted as the rate of myosin transition from the weakly to strongly bound state or the (apparent) rate of myosin force production (Zhao & Kawai, 1993). The frequency portion of process C (2π*c*) represents the cross‐bridge detachment rate (Palmer et al., 2007). The 2π*c*/2π*b* ratio is the ratio between the rate of detachment and the rate of force production. From the sinusoidal analysis, work (in joules per metre cubed) was calculated as: work = πft(−E_v_)(L_amp_)^2^, where f is the frequency of the length perturbations (in hertz), t is the time needed to perform the length perturbations (in seconds), E_v_ is the viscous modulus (N/mm^2^), and the fractional change in length (L_amp_) is 0.0005% of fibre length. Of note, work is positive when the viscous modulus plot is negative.

### 2.9 Identification of MHC isoforms

After sinusoidal analysis measurements, single fibres were placed in 30 µL loading buffer, heated for 2 min at 65°C, and stored at −80°C until determination of MHC isoform composition by SDS‐PAGE to identify fibre type, as described by Miller et al. (2010).

### 2.10 Statistical analyses

All data are reported as the mean ± SD, and differences were considered significant at *P* ≤ 0.05. Given that there were multiple observations within the same participant (i.e., multiple fibres per participant), main effects for each fibre type were determined using a repeated‐measures linear mixed model, with a random effect to account for grouping observations within participants, as reported previously (Miller et al., 2013; Momb et al., 2022), and a repeated effect to account for the between‐participant factor (control, high P_i_, low pH and fatigue). If a main effect was noted, Fisher's least‐significant difference *post hoc* test was performed to determine pairwise differences between the experimental conditions. All analyses were conducted using IBM SPSS Statistics for Windows v.25.0 (IBM, Armonk, NY, USA).

## 3 RESULTS

### 3.1 Participant characteristics

Thirteen older adults (8 female) aged 70.8 ± 1.9 years (range, 66–76 years) were included in this study. The participants had a height of 171.4 ± 4.2 cm (range, 160–178 cm) body mass of 75.2 ± 4.5 kg (range, 51.8–98.7 kg) and body mass index of 25.6 ± 1.4 kg/m^2^ (range, 18.3–31.8 kg/m^2^). Daily step count averaged 6481 ± 2342 (range, 3680–11 181), weekly moderate‐to‐vigorous activity was 282 ± 111 min (range, 124–523 min), with daily activity counts of 417 ± 58 a.u. (range, 350–539 a.u.).

### 3.2 Cellular‐level outcomes

**FIGURE 2:**
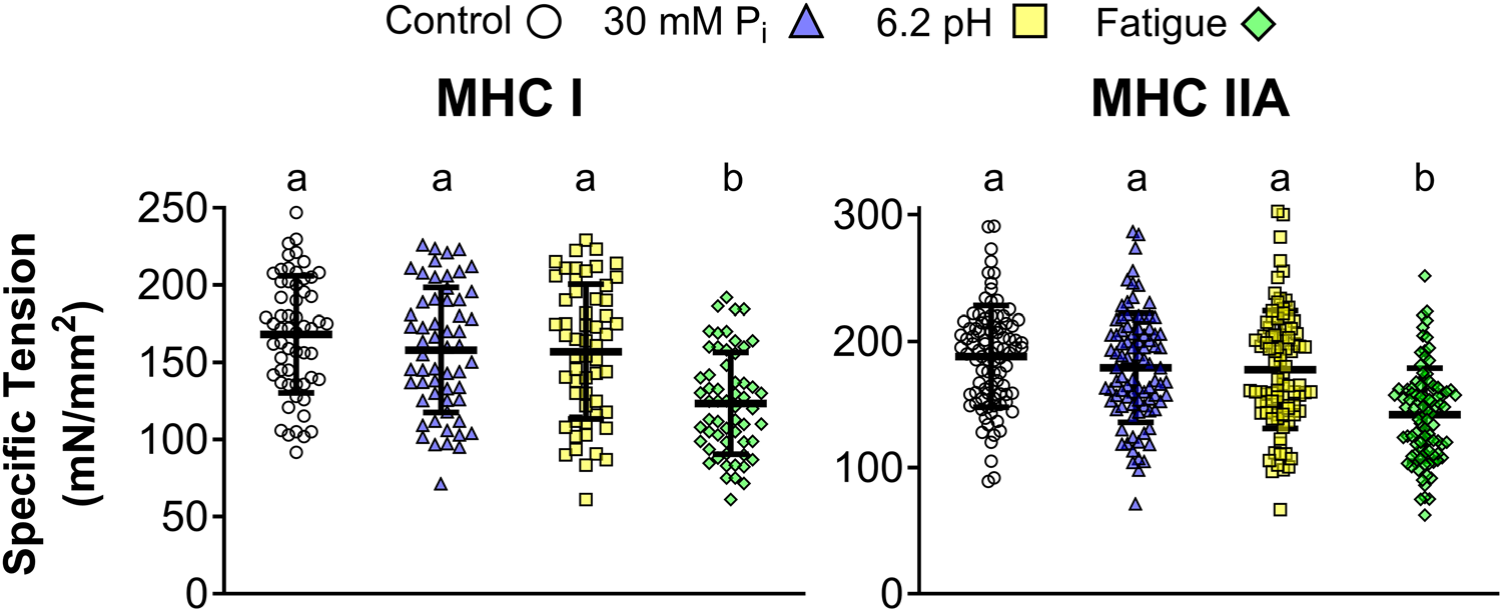
Single‐fibre maximum Ca^2+^‐activated (pCa 4.5) specific tension by MHC isoform for control, high P_i_, low pH and fatigue conditions at 37°C. Data are presented as the mean ± SD, with individual fibres as data points. Where condition effects were observed, different letters above the data identify significant (*P* ≤ 0.05) pairwise differences between conditions within each fibre type. The *P*‐values are reported in the main text (see Results). The number of fibres (*n*) tested for each condition is as follows: MHC I, *n* = 58; and MHC IIA, *n* = 92. Abbreviations: MHC, myosin heavy chain; P_i_, inorganic phosphate.

Data are reported for MHC I (*n* = 58) fibres and MHC IIA (*n* = 92) fibres. Seven fibres (4.6% of the total number of fibres examined) were excluded from analysis, with two expressing MHC I and five expressing MHC IIA, owing to specific tension loss of >10% or sinusoidal analysis data that could not be fitted adequately to the six‐parameter model. Statistics were not completed on MHC I/IIA (*n* = 2), IIA/IIX (*n* = 13), I/IIA/IIX (*n* = 1) or IIX (*n* = 0) owing to inadequate sample sizes. Manipulating P_i_ or pH independently did not change specific tension compared with control conditions in MHC I (P_i_, *P* = 0.0942; pH, *P* = 0.0655) or IIA fibres (P_i_, *P* = 0.413; pH, *P* = 0.260; Figure 2). In contrast, altering both P_i_ and pH in solution (fatigue) reduced specific tension in MHC I (20%, *P* < 0.001) and IIA (26%, *P* < 0.001) fibres compared with control conditions. These results indicate that an interdependent effect occurs with the combination of high P_i_ and low pH in comparison to either condition alone, and that this effect is robust at physiological temperature. The percentage change in specific tension from the beginning (initial activation) to the end of the condition (immediately after sinusoidal analysis) provides a measure of fibre stability and was decreased to a similar extent for both fibre types in all conditions (Figure 1b).

### 3.3 Molecular‐level cross‐bridge mechanical analysis

**FIGURE 3:**
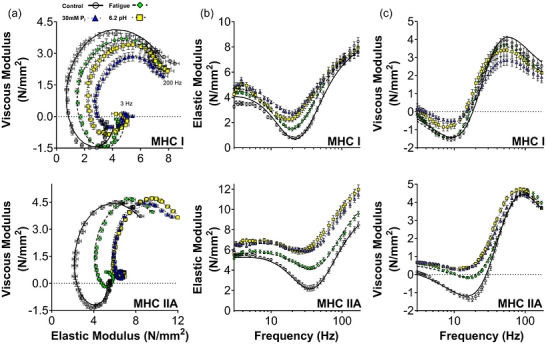
Single skeletal muscle fibre maximal Ca^2+^‐activated sinusoidal analysis, showing Nyquist plots and Bode plots across frequency by MHC isoform for control, high P_i_, low pH and fatigue conditions at 37°C. The mean ± SD for each frequency is shown. Lines represent the fits from the six‐parameter model. The number of fibres (*n*) tested for each condition is as follows: MHC I, *n* = 58; and MHC IIA, *n* = 92. Abbreviations: MHC, myosin heavy chain; P_i_, inorganic phosphate.

**FIGURE 4:**
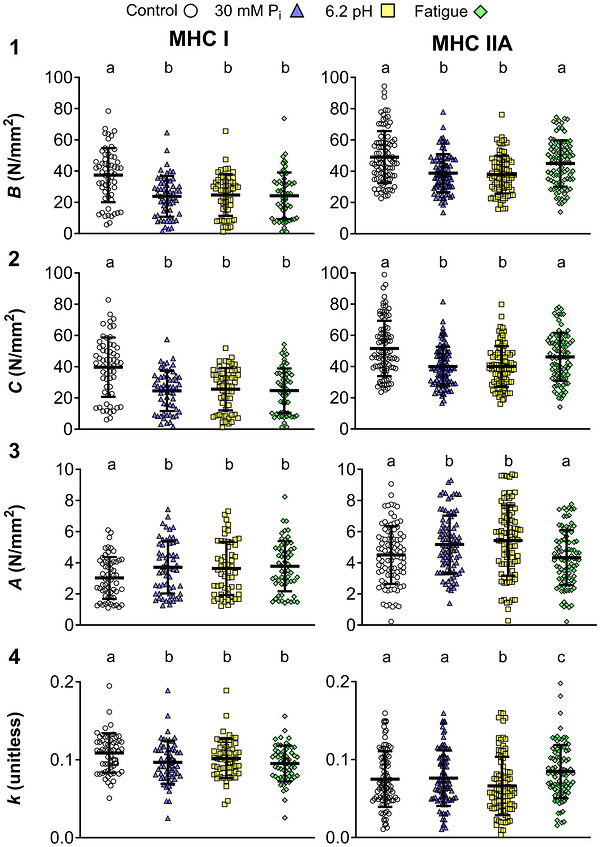
Single‐fibre maximum Ca^2+^‐activated (pCa 4.5) sinusoidal analysis model, showing mechanical parameters by MHC isoform for control, high P_i_, low pH and fatigue conditions at 37°C. (a,b) Parameters *B* (a) and *C* (b) are proportional to the number of myosin heads strongly bound to actin and/or cross‐bridge stiffness. (c,d) Parameters *A* (c) and *k* (d) represent the underlying stiffness of the lattice structure and the attached myosin heads in series. Data are presented as the mean ± SD, with individual fibres as data points. Where condition effects were observed, different letters above the data identify significant (*P* ≤ 0.05) pairwise differences between conditions within each fibre type. The *P*‐values are reported in the main text (see Results). The number of fibres (*n*) tested for each condition is as follows: MHC I, *n* = 58; and MHC IIA, *n* = 92. Abbreviations: MHC, myosin heavy chain; P_i_, inorganic phosphate.

**FIGURE 5:**
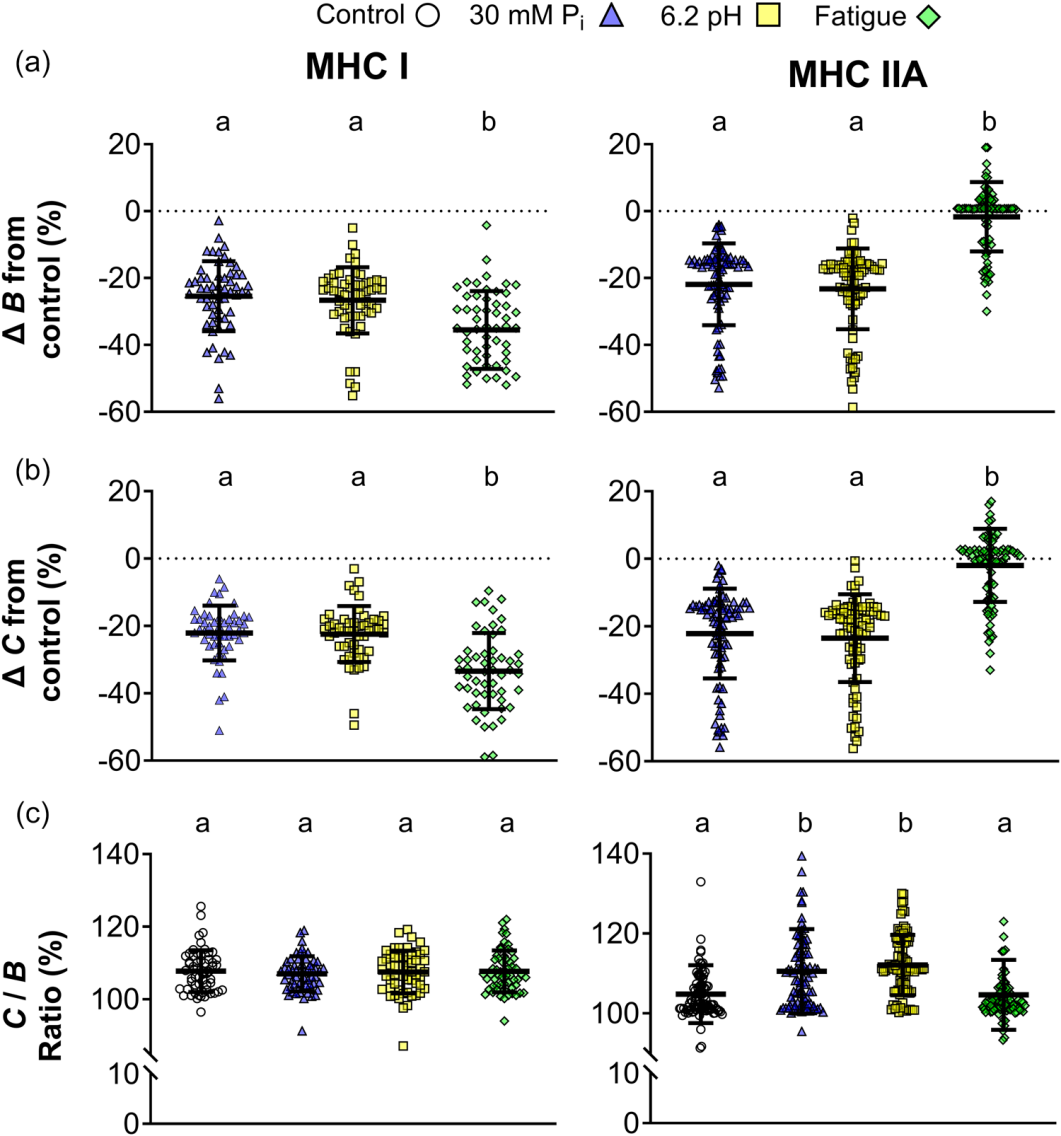
Relative changes in sinusoidal analysis model parameters *B* and *C* by MHC isoform for control, high P_i_, low pH and fatigue conditions at 37°C. (a,b) Parameters *B* and *C* are proportional to the number of myosin heads strongly bound to actin and cross‐bridge stiffness during maximum Ca^2+^ activation, respectively. (c) *C*/*B* is the ratio of parameter *C* (the magnitude of work absorption) and parameter *B* (the magnitude of work production). Single‐fibre data points are overlayed on top of bars. Data are presented as the mean ± SD, with individual fibres as data points. Where condition effects were observed, different letters above the data identify significant (*P* ≤ 0.05) pairwise differences between conditions within each fibre type. The *P*‐values are reported in the main text (see Results). Horizontal dotted lines in (a) and (b) indicate zero relative change in *B* or *C*. The number of fibres (*n*) tested for each condition is as follows: MHC I, *n* = 58; and MHC IIA, *n* = 92. Abbreviations: MHC, myosin heavy chain; P_i_, inorganic phosphate.

**FIGURE 6:**
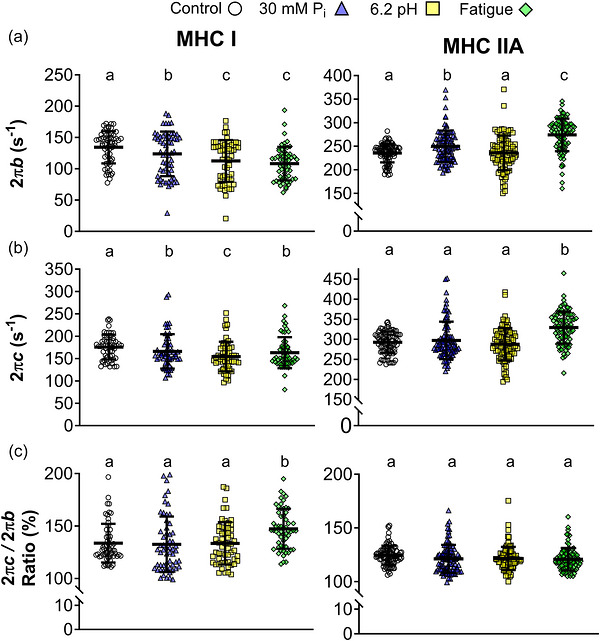
Single‐fibre maximum Ca^2+^‐activated (pCa 4.5) sinusoidal analysis model kinetic parameters and ratios by MHC isoform for control, high P_i_, low pH and fatigue conditions at 37°C. (a) 2π*b* is the rate of myosin transition between the weakly and strongly bound states. (b) 2π*c* is the rate of myosin detachment from actin. (c) 2π*c*/2π*b* is the ratio of the kinetic rate constants of cross‐bridge detachment and cross‐bridge recruitment. Data are presented as the mean ± SD, with individual fibres as data points. Where condition effects were observed, different letters above the data identify significant (*P* ≤ 0.05) pairwise differences between conditions within each fibre type. The *P*‐values are reported in the main text (see Results). The number of fibres (*n*) tested for each condition is as follows: MHC I, *n* = 58; and MHC IIA, *n* = 92. Abbreviations: MHC, myosin heavy chain; P_i_, inorganic phosphate.

Nyquist plots of the elastic and viscous moduli at each oscillation frequency were created for the various conditions examined in MHC I and IIA fibres (Figure 3a). Each fibre had a Nyquist plot and was fitted using Equation 1, then fitted to the six‐parameter model representing myosin–actin cross‐bridge mechanics and kinetics (Figures 4, 5, 6). The Nyquist plots can be broken down into Bode plots representing the elastic and viscous moduli across each frequency (Figure 3b,c). The mechanical analysis, involving parameters *B* and *C*, indicated that the number and/or stiffness of strongly bound myosin–actin cross‐bridges were reduced with greater P_i_ or lower pH in MHC I fibres (all *P* < 0.001) and MHC IIA fibres (all *P* < 0.001; Figure 4a,b). The myofilament properties analysis showed that parameter *A* increased and *k* decreased in conditions of high P_i_ or low pH in MHC I (parameter *A*, P_i_, *P* = 0.00258 and pH, *P* = 0.00908; *k*, P_i_, *P *= 0.00375 and pH, *P* = 0.0413) and IIA fibres (parameter *A*, P_i_, *P* = 0.0182 and pH, *P* < 0.001; *k*, pH, *P* = 0.00378), with the exception that *k* remained unchanged for high P_i_ in MHC IIA (*P* = 0.385; Figure 4c,d). These alterations to *A* and *k* indicate that P_i_ and pH each independently increase the myofilament stiffness of MHC I and IIA fibres.

In fatigue conditions, MHC I fibres displayed reductions in *B* and *C* compared with control (*P* < 0.001), with a concomitant increase in *A* (*P* = 0.00117) and decrease in *k* (*P* < 0.001; Figure 4, left panels), similar to the MHC I response to high P_i_ or low pH. To understand why specific tension dropped in MHC I with fatigue, but not with high P_i_ or low pH individually, the percentage change of *B* and *C* from control, or Δ*B* and Δ*C*, was determined for each condition because parameters *B* and *C* inherently have a high level of variability. Expression as a percentage change shows the consistency and magnitude of change within individual fibre experiments. The fatigue condition produced greater decreases in Δ*B* and Δ*C* (33%–35%, both *P* < 0.001) relative to the independent manipulations of P_i_ and pH (*P* = 0.411–0.519), which showed similar reductions in Δ*B* and Δ*C* (21%–27%, *P* = 0.524–0.595; Figure 5a b, left panels). These results indicate that the reduction in specific tension with fatigue is attributable to fewer strongly bound myosin–actin cross‐bridges and/or their stiffness compared with P_i_ or pH alone. The percentage change was examined for the other parameters, *A* and *k*, in addition to specific tension, but parameters *B* and *C* were the only variables that had significantly different responses in these measures across conditions.

In MHC IIA fibres, fatigue conditions increased *k* (*P* < 0.001), with no change in parameters *B* (*P* = 0.285), *C* (*P* = 0.0834) or *A* (*P* = 0.381; Figure 4, right panels), suggesting that the reduction in specific tension with fatigue was attributable to fibres becoming more viscous or work‐absorbing. The fatigue condition did not significantly change Δ*B* or Δ*C* relative to the control condition, as expected because there was no change in *B* and *C*, and was higher than the P_i_ and pH conditions (*P* < 0.001; Figure 5a,b, right panels). The independent manipulations of P_i_ and pH decreased Δ*B* and Δ*C* to a similar extent (21%–24%; *P *= 0.387–0.482), as expected owing to their lower values of *B* and *C*.

The ratio between the magnitude of the work‐absorbing, or positive viscous modulus, process C (*C*) and the work‐producing, or negative viscous modulus, process B (*B*) provides potential insight into fatigue‐induced alterations in contractile properties. The *C*/*B* ratio was unchanged in MHC I fibres in any condition (*P* = 0.475–0.944; Figure 5c, left panel). In contrast, in MHC IIA fibres this ratio was increased for P_i_ (*P* < 0.001) and pH (*P* < 0.001) alone but not combined (*P* = 0.904; Figure 5c, right panel). A higher *C*/*B* ratio indicates that the work‐absorbing processes became relatively larger, a result that will become more relevant when examining the changes in oscillatory work production.

### 3.4 Molecular‐level cross‐bridge kinetic analysis

In MHC I fibres, the rates of myosin force production (2π*b*; P_i_, *P* = 0.0234; pH, *P* < 0.001; fatigue, *P* < 0.001) and cross‐bridge detachment (2π*c*; P_i_, *P *= 0.0453; pH, *P* < 0.001; fatigue, *P* = 0.00848) slowed in all conditions compared with the control condition (Figure 6a,b, left panels). The fatigue and low pH conditions had the slowest 2π*b*, whereas 2π*c* was most altered by low pH alone. This last result suggests that, in fatigue, high P_i_ has a moderating effect on the slowing of cross‐bridge detachment owing to acidosis in MHC I fibres.

In MHC IIA fibres, high P_i_ increased 2π*b* (*P* < 0.001) but not 2π*c* (*P* = 0.380). Low pH did not alter cross‐bridge attachment kinetics (*P* = 0.827) or detachment kinetics (*P* = 0.270). In contrast, fatigue increased both 2π*b* (*P* < 0.001) and 2π*c* (*P* < 0.001), indicating that P_i_ and pH have an interdependent effect on cross‐bridge kinetics, and an opposite response compared with the slowing of kinetics observed in MHC I fibres. These changes in cross‐bridge kinetics did not alter their ratios (2π*c*/2π*b*, *P *= 0.796–0.952), except for fatigue in MHC I fibres, where this ratio increased (*P* < 0.001; Figure 6c), suggesting that the slowing of cross‐bridge detachment rate exceeded the slowing of the rate of force production.

### 3.5 Analysis of oscillatory work

**FIGURE 7:**
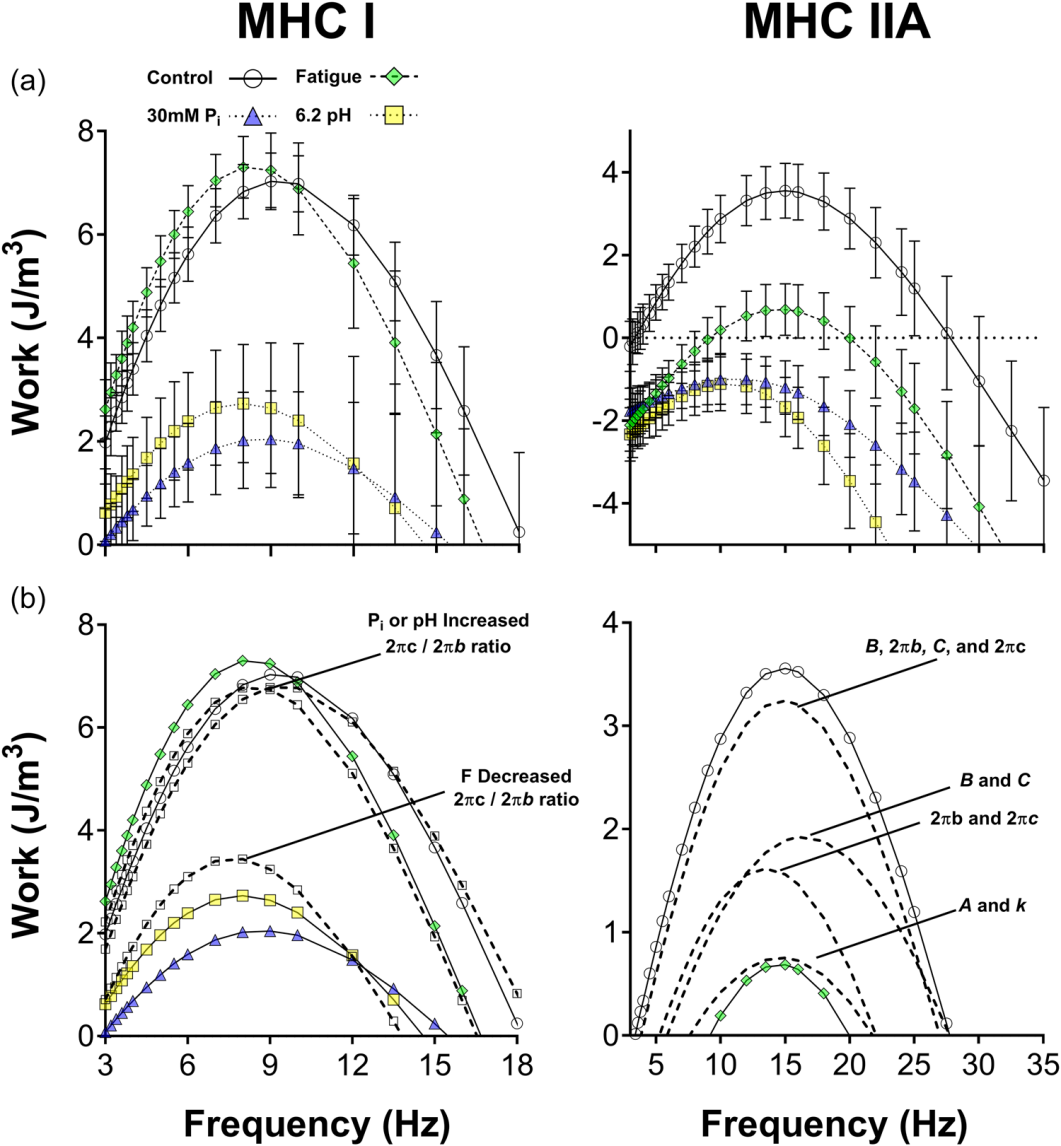
Single‐fibre maximum Ca^2+^‐activated (pCa 4.5) oscillatory work versus frequency by MHC isoform for control, high P_i_, low pH and fatigue conditions at 37°C. (a) Oscillatory work for control, low pH, high P_i_ and fatigue conditions by MHC isoform. (b) Models altering the 2π*c*/2π*b* ratio (left, MHC I) or the different model parameters (right, MHC IIA) and their impact on work versus frequency. Data are presented as the mean ± SD. Models are compared with initial conditions of control, fatigue, high P_i_ and low pH for MHC I and with control and fatigue for MHC IIA. The horizontal dotted line in the upper right‐hand panel indicates zero work. The number of fibres (*n*) tested for each condition is as follows: MHC I, *n* = 58; and MHC IIA, *n* = 92. Abbreviations: F, fatigue; MHC, myosin heavy chain; P_i_, inorganic phosphate.

Oscillatory work is a direct measurement of viscous modulus and was examined to understand how the six model parameters impact cross‐bridge function when summed together. Oscillatory work was reduced from control in high P_i_ and low pH conditions for both MHC I and IIA fibres (Figure 7a; Table 2), becoming negative in MHC IIA fibres. Reduced work was attributable to the decreased *B* and *C* discussed above and shown in Figure 4. The larger decrease in work in MHC IIA compared with MHC I fibres was probably caused by the increase in *C*/*B* in the individual high P_i_ and low pH conditions (Figure 5), because increasing the proportion of work absorbed will decrease the overall work produced. The frequency of peak work was reduced for pH but not P_i_ in MHC I fibres (Table 2), probably owing to the greater slowing of cross‐bridge kinetics with pH compared with P_i_ (Figure 6a,b, left panels). In MHC IIA fibres, the frequency of peak work was reduced for both P_i_ and pH conditions, probably owing to an increase in *C*/*B* (Figure 5c), because cross‐bridge kinetics were either faster or unchanged (Figure 6a,b, right panels), and the 2π*c*/2π*b* ratio was unchanged (Figure 6c, right panels) compared with control.

**TABLE 2:** Summary of oscillatory work for MHC I and MHC IIA fibres.

Fibre type	Condition	Peak work (mJ/m^3^)	*P*‐value	Frequency of peak work (Hz)	*P*‐value
MHC I	Control	7.03 ± 0.07^a^	–	9.92 ± 0.61^a^	–
	P_i_ = 30 mM	3.19 ± 0.12^b^	<0.001	9.65 ± 0.52^a^	0.593
	pH = 6.2	4.38 ± 0.16^c^	<0.001	8.28 ± 0.46^b^	<0.001
	Fatigue	7.31 ± 0.09^a^	0.358	8.07 ± 0.49^b^	<0.001
MHC IIA	Control	6.32 ± 0.16^a^	–	15.23 ± 0.42^a^	–
	P_i_ = 30 mM	−1.55 ± 0.19^b^	<0.001	12.43 ± 0.54^b^	<0.001
	pH = 6.2	−1.11 ± 0.09^b^	<0.001	10.11 ± 0.34^c^	<0.001
	Fatigue	0.69 ± 0.12^c^	<0.001	15.22 ± 0.73^a^	0.442

*Note*: Data are presented as the mean ± SD. Superscript letters indicate significant pairwise differences between conditions, with *P*‐values compared with control.

Abbreviations: MHC, myosin heavy chain; P_i_, inorganic phosphate.

Fatigue caused fibre type‐specific effects such that in MHC I fibres a minimal change in oscillatory work was noted, but with a decreased frequency of maximal work, whereas in MHC IIA fibres the work was reduced, with no change in frequency (Figure 7a; Table 2). MHC I fibres in fatiguing conditions showed similar changes in most parameters compared with P_i_ and pH alone, except for cross‐bridge kinetics, especially 2π*c*/2π*b*, which was increased with fatigue (147%) compared with P_i_ and pH alone (132%–133%; Figure 6c, left panel). To determine the impact of 2π*c*/2π*b* on work production, we created a hypothetical scenario, in which the 2π*c* fitted parameter values were increased for P_i_ and pH alone such that their 2π*c*/2π*b* values were increased to the fatigue value of 150% (Figure 6c, left panel), while the remaining parameters (2π*b*, *B*, *C*, *A* and *k*) were left unchanged. By increasing only one parameter, 2π*c*, work curves for P_i_ and pH resembled the work curve for fatigue (Figure 7b, left panel). Likewise, if the 2π*c* value for fatigue was reduced such that the 2π*c*/2π*b* value was decreased to the approximate P_i_ and pH alone value of 130%, while the remaining parameters (2π*b*, *B*, *C*, *A* and *k*) were left unchanged, this work curve resembled the work curve for P_i_ and pH alone (Figure 7b, left panel).

MHC IIA fibres in fatiguing conditions had reduced peak work compared with control, with the major changes to the six‐parameter model being a higher *k* and faster cross‐bridge kinetics, 2π*b* and 2π*c*. To determine the impact of altering these parameters on oscillatory work production, the fitted parameter values from control conditions were substituted into the fatiguing conditions for *A* and *k* alone, 2π*b* and 2π*c* alone, *B* and *C* alone, or *B*, *C*, 2π*b* and 2π*c* combined (Figure 7b, right panel). By increasing only *A* and *k*, work production was modestly increased, indicating that changes to myofilament stiffness are not large contributors to the decrease in work in MHC IIA fibres. By altering 2π*b* and 2π*c* or *B* and *C* independently, work is shifted substantially higher relative to fatigue but does not completely explain the reduction in work with fatigue. Although the mean values of *B*, *C* and *B*/*C* were not statistically different between control and fatigue groups (Figures 4a,b and 5c, right panels), the slight alterations to *B*/*C* from 107% to 105% in this model made fibres less work‐absorbing and more work‐producing. Thus, when altering *B* and *C* in conjunction with 2π*b* and 2π*c*, work production is returned almost to control conditions.

## 4 DISCUSSION

We quantified cellular and molecular contractile function at 37°C in single human skeletal muscle fibres, with the manipulation of P_i_ and pH independently and combined. Our results show that specific tension is reduced by distinct molecular mechanisms in slow‐ versus fast‐contracting fibres when manipulating P_i_ (30 mM) and pH (6.2) in tandem, termed fatigue. In MHC I fibres, we observed a distinct reduction in the number of strongly bound cross‐bridges and/or stiffness, whereas MHC IIA fibres had greater myofilament viscosity, leading to more work absorption. Cross‐bridge kinetics also responded differently by fibre type, with MHC I fibres slowing in all three conditions (high P_i_, low pH and fatigue) but MHC IIA fibres quickening with high P_i_ and fatigue. These alterations to molecular mechanics and kinetics led to reduced oscillatory work in both MHC I and IIA fibres when independently altering P_i_ or pH. But with fatigue, oscillatory work was either returned to control values for MHC I, or closer to control values in MHC IIA fibres. The mechanisms behind changes to work with fatigue were also fibre type specific, with MHC I fibres increasing the ratio of the cross‐bridge detachment rate to the rate of myosin force production, whereas MHC IIA fibres had alterations to work production and absorption processes (processes B and C). These findings highlight that: (1) MHC I and IIA fibres have a similar reduction in force in response to fatigue; but (2) the molecular mechanisms leading to reductions in force, and potential alterations to contractile velocities, are fibre type specific at physiological temperatures.

### 4.1 Effects of fatigue on force production

Single‐fibre specific tension is the total force established by the number of strongly bound cross‐bridges, the force per cross‐bridge and the stiffness of the myofilaments (Brenner et al., 2012; Capitanio et al., 2006; Linari et al., 2004). Reductions in force production with fatigue are thought to be attributable to fewer force‐generating cross‐bridges and/or reductions in force per cross‐bridge (Foster et al., 2025; Nelson et al., 2014; Nocella et al., 2011). This agrees well with our results in MHC I fibres, where the lowering of specific tension with fatigue occurs along with a reduction in the number of strongly bound cross‐bridges and/or their stiffness. In MHC IIA fibres, several mechanisms might explain the loss of force production, with no change in the number of strongly bound cross‐bridges and/or stiffness. Previous work from our laboratory has examined fibres in rigor (no ATP) (Foster et al., 2025), meaning that myosin heads are not going through the cross‐bridge cycle and that >94% of the myosin heads consistently remain strongly bound (Cooke & Franks, 1980; Lovell et al., 1981). In rigor conditions, MHC IIA fibres in fatigue showed a decreased force per cross‐bridge, without a reduction in cross‐bridge stiffness compared with control conditions (Foster et al., 2025). Thus, one mechanism to explain lower force in MHC IIA fibres is that the number of strongly bound cross‐bridges and their stiffness might remain similar in control and fatiguing conditions, resulting in similar values of *B* and *C* (Figure 4a,b, right panel), but force per cross‐bridge decreases with fatigue, which would reduce overall force production. Another mechanism might be the observed increase in myofilament viscosity with fatigue, which would increase energy absorption, potentially reducing force production. Previous work has identified an inverse relationship between cross‐bridge lifetime and *k* (Palmer et al., 2013; Wang et al., 2013) such that faster cross‐bridge kinetics (increases in 2π*b* and 2π*c*; Figure 6a,b, right panel) increase myofilament viscosity (increase in *k*; Figure 4d, right panel). Alternatively, the reduced force‐generating capacity per cross‐bridge or changes in other myofilament proteins, such as titin, might be increasing myofilament viscosity. These findings highlight potential differences between the effects of fatigue on slow‐ and fast‐contracting fibres, with the precise mechanism(s) behind the loss of force production needing to be determined in future experiments.

### 4.2 Effects of fatigue on cross‐bridge kinetics

Our results at 37°C show that cross‐bridge kinetics responded to fatigue differently by fibre type, with MHC I fibres slowing and MHC IIA fibres becoming faster (Figure 6a,b; Table 3). In a two‐state model, where myosin is either strongly bound to actin in a force‐producing state or in a detached non‐force‐producing state, the number of strongly bound cross‐bridges is equal to the total number of available cross‐bridges (*N*) multiplied by the fraction of time that a cross‐bridge is formed [*f*_app_/(*f*_app_ + *g*_app_)], where *f*_app_ and *g*_app_ are the transitions from the detached to strongly bound states and from the strongly bound to detached states (Brenner, 1988). In our experiments, *N* remains constant between the various conditions because the total number of available cross‐bridges remains unchanged. Our rate of cross‐bridge detachment (2π*c*) should be equivalent to *g*_app_ (Palmer et al., 2007). Our rate of myosin force production (2π*b*), or the rate of myosin transition from the weakly to the strongly bound state (Zhao & Kawai, 1993), comprises a portion of *f*_app_, but the detached to weakly bound transition cannot be measured using our sinusoidal analysis technique. In MHC IIA fibres, the faster 2π*c*, or *g*_app_, with fatigue should decrease the number of strongly bound cross‐bridges based upon the equation above, while the faster 2π*b* should do the opposite. Given that the number of strongly bound heads and their stiffness remain similar between control and fatiguing conditions in MHC IIA fibres, this implies that the changes in *f*_app_ are offset by the changes in *g*_app_. In MHC I fibres the opposite occurs, in that the slower 2π*c* with fatigue should increase the number of strongly bound cross‐bridges, while the slower 2π*b* should decrease the number of strongly bound cross‐bridges. Our previous work using rigor conditions showed that both cross‐bridge stiffness and force per cross‐bridge were decreased with fatigue in MHC I fibres (Foster et al., 2025), meaning that the reduced *B* and *C* with fatigue could be attributable to loss of cross‐bridge stiffness and/or the number strongly bound cross‐bridges. This suggests that if fatigue decreases *B* and *C* by cross‐bridge stiffness alone, the changes in *f*_app_ are offset by the changes in *g*_app_, or by the number of strongly bound cross‐bridges alone, *f*_app_ is more affected by fatigue than *g*_app_. Our findings quantify fibre type‐specific kinetic mechanisms of fatigue in human skeletal muscle at 37°C, thus advancing our understanding of metabolite‐based muscle fatigue in vivo.

**TABLE 3:** Summary of independent and combined effects of reduced pH and increased inorganic phosphate on cellular and molecular contractile function.

Variable	30 mM P_i_		6.2 pH		Fatigue	
	I	IIA	I	IIA	I	IIA
Specific tension (mN/mm^2^)	↔	↔	↔	↔	↓↓	↓↓
Strongly bound cross‐bridges (N/mm^2^)	↓↓	↓↓	↓↓	↓↓	↓↓↓	↔
Myofilament stiffness	↑	↑↑	↑	↑↑	↑	↓↓
2π*b* (s^−1^)	↓	↑	↓↓	↔	↓	↑↑
2π*c* (s^−1^)	↓	↔	↓↓	↔	↓	↑
Peak work (J/m^3^)	↓↓↓	↓↓↓↓	↓↓	↓↓↓↓	↔	↓↓↓

*Note*: Strongly bound cross‐brides are derived from parameters *B* and *C* and myofilament stiffness from parameters *A* and *k*; 2π*b* is the rate of cross‐bridge attachment, and 2π*c* is the rate of cross‐bridge detachment. Peak oscillatory work is derived from sinusoidal analysis at the lowest value of the viscous modulus.

Symbols: ↑, increase; ↔, no change, ↓, decrease; number of arrows signifies magnitude of response, with one arrow indicating 10%–20% change, two arrows 20%–50%, three arrows >50%–100%, and four arrows >100%.

Abbreviation: P_i_, inorganic phosphate.

**FIGURE 8:**
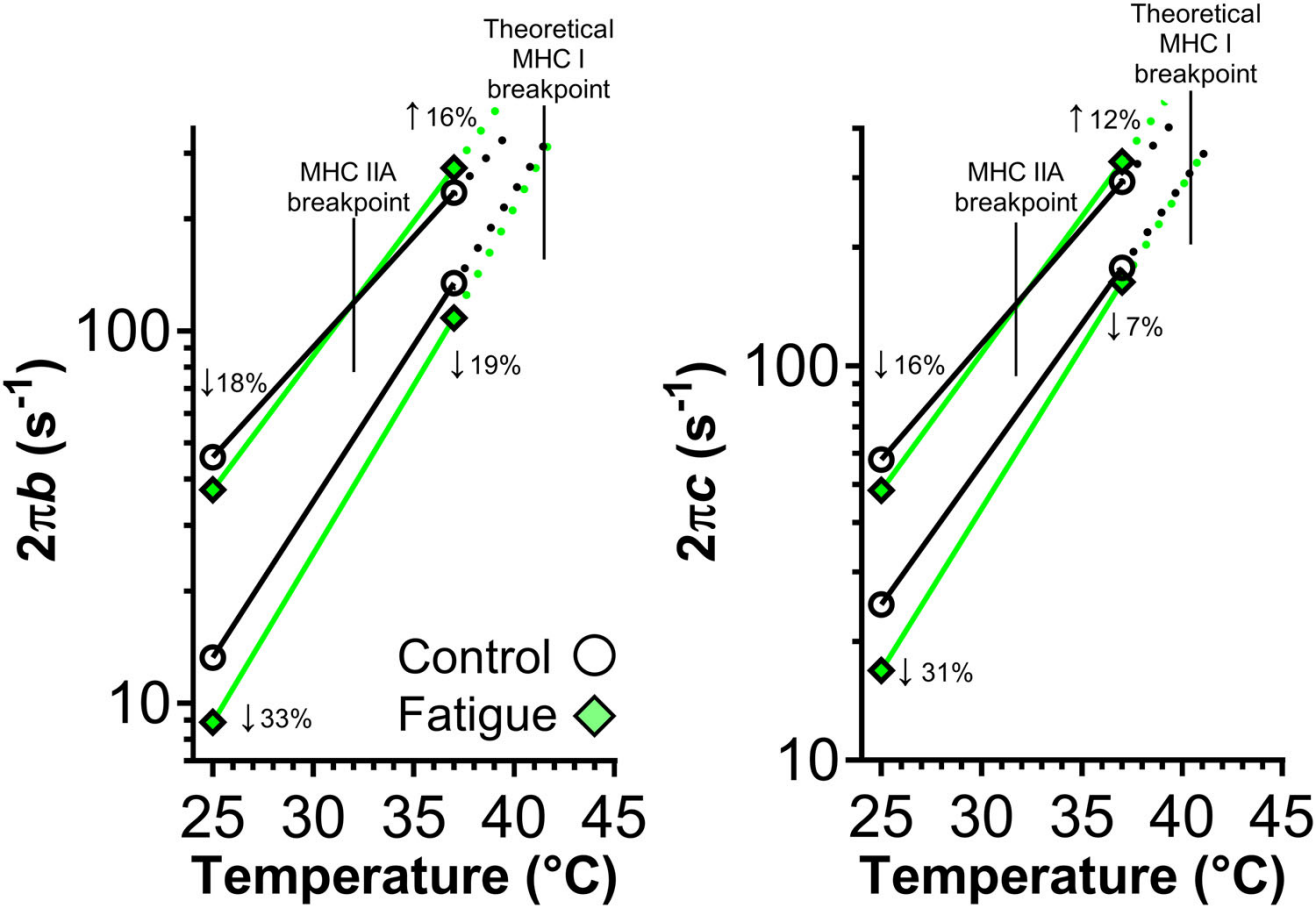
Single‐fibre maximum Ca^2+^‐activated (pCa 4.5) sinusoidal analysis model kinetic parameters by MHC isoform for control and fatigue conditions at 25 and 37°C. Data for 2π*b* and 2π*c* at 25°C are from our previously published work (Foster et al., 2025) and at 37°C from this work. In MHC I and IIA fibres, cross‐bridge kinetics are slower with fatigue than control at 25°C and increase more with temperature in the fatigue condition compared with control. We hypothesize that this causes MHC IIA fibres to have a breakpoint in 2π*b* and 2π*c* at ∼32°C, where fatigue and control cross‐bridge kinetics are equal, and for temperatures of >32°C fatigue cross‐bridge kinetics will be greater than control. We hypothesize that at temperatures between ∼40‐45°C, MHC I fibres will display a similar breakpoint for fatigue and control cross‐bridge kinetics. A linear relationship between the logarithm of 2π*b* and 2π*c* with temperature is assumed based on previous work by others in rabbit soleus and psoas tissue (Wang & Kawai, 2001: fig. 4). Abbreviation: MHC, myosin heavy chain.

Prior studies of fatigue in human MHC I and IIA fibres have shown slower cross‐bridge kinetics, specifically a reduced rate of force development (*k*_tr_) at 15°C (Sundberg et al., 2018) and decreased 2π*b* and 2π*c*, observed as increased myosin attachment time [*t*_on_, which equals (2π*c*)^−1^ (Palmer et al., 2013)], at 25°C (Foster et al., 2025). The opposite fatigue response in MHC IIA fibres at 37°C in the present study, the increased 2π*b* and 2π*c*, is likely to be attributable to the higher temperature, because previous work at 25°C was performed in the same laboratory using identical equipment and also in tissue from healthy older adults (Foster et al., 2025). Part of the explanation for this might be that when transitioning from 25°C to 37°C, 2π*b* and 2π*c* values increase greatly in a fibre type‐specific manner, being 6.7‐ to 9.4‐fold higher for MHC I fibres and 4.7‐ to 4.8‐fold higher for MHC IIA fibres for control conditions (Momb et al., 2025). The faster cross‐bridge kinetics at higher temperatures in MHC I fibres dampens their response to fatigue as cross‐bridge kinetics slow by 31%–33% at 25°C (Foster et al., 2025) but by only 7%–19% at 37°C (Figure 8). MHC IIA fibres experience a breakpoint between 25°C and 37°C as fatigue slows cross‐bridge kinetics by 20%–22% at 25°C (Foster et al., 2025) but quickens cross‐bridge kinetics by 12%–16% (Figure 8). Thus, for both fibre types, moving from 25°C to 37°C quickens cross‐bridge kinetics, but more so for fatigue than control conditions, with MHC IIA fibres having a breakpoint (Figure 8). In theory, if temperature increased beyond 37°C, there would be a breakpoint for MHC I fibres where fatigue conditions cause cross‐bridge kinetics to become quicker (Figure 8). One potential mechanism behind the differential fatigue response is that the MHC isoforms might react differently to load, because we have observed that fast‐contracting fibres (MHC IIA, IIX and IIB) that are quickly stretched during fatigue have a large stretch activation response, whereas slow‐contracting fibres (MHC I) are almost unresponsive (Woods et al., 2025). Notably, conditions that produce high resistive loads on the myosin, such as our isometrically contracting fibres and the mini‐ensemble laser trap assay (Woodward & Debold, 2018), show that fast‐contracting myosin has a faster rate of detachment with fatigue. The response to load in these fibre types might be attributable to differences in a variety of factors, including isoform differences in the myosin head structure or function, regulatory protein differences (thin filament proteins, titin, myosin binding protein‐C, etc.) and fibre type‐specific post‐translational modifications, which are mechanisms that should be examined in future work.

In summary, our findings of slower cross‐bridge kinetics in MHC I fibres agree well with previous human work (Foster et al., 2025; Sundberg et al., 2018), and our findings of faster cross‐bridge kinetics in MHC IIA fibres agree with previous animal work (Woodward & Debold, 2018). These differences in fibre‐type response might be attributable to their response to load, which should be evaluated using the appropriate techniques, such as the laser trap assay.

### 4.3 Summary of effects of fatigue

Based on the present study and our previous work (Foster et al., 2025), force production in MHC I fibres is reduced in fatigue owing to fewer strongly bound cross‐bridges and/or decreased force per cross‐bridge, potentially attributable, in part, to decreased cross‐bridge stiffness. In MHC IIA fibres, force production is reduced in fatigue owing to either a decreased force per cross‐bridge and/or increased viscosity of the myofilaments or cross‐bridges. Notably, decreased force per cross‐bridge is a potential explanation in both fibre types for the loss of force with fatigue, a possibility also suggested by others (Nelson et al., 2014). Cross‐bridge kinetics responded to fatigue differently by fibre type, with MHC I fibres slowing and MHC IIA becoming faster. These changes, especially in 2π*c* [given that myosin attachment time or *t*_on_ equals (2π*c*)^−1^ (Palmer et al., 2013)] should have the opposite effects on contractile velocity (Miller & Toth, 2013; Tyska & Warshaw, 2002), slowing in MHC I fibres and increasing in MHC IIA fibres, assuming that the cross‐bridges respond in a similar manner to fatigue in the lower‐load conditions during contraction compared with the high‐load conditions during isometric conditions, such as used in this and our previous work (Foster et al., 2025).

### 4.4 Effects of P_i_ or pH on force production

In the present study, manipulating P_i_ or pH independently caused no change in specific tension in either MHC I or IIA at 37°C, owing to a counterbalancing effect of a decrease in strongly bound cross‐bridges but a concomitant increase in myofilament stiffness (Figures 2 and 4). These findings indicate that stiffness of the thick and/or thin filament is increasing, potentially leading to higher force transfer through the myofilaments and causing specific tension to be similar across conditions (control, high P_i_ or low pH). Prior work highlights a temperature‐dependent effect when altering P_i_ or pH independently, whereby force production decreases markedly at 15°C, and depression of force is only modest at 30°C or higher (Coupland et al., 2001; Debold et al., 2004; Knuth et al., 2006; Westerblad et al., 1997), aligning well with our results of no change at 37°C (Figure 2). Reductions in force at the cross‐bridge level with elevated P_i_ have been proposed to occur owing to early cross‐bridge detachment (Marang et al., 2025), resulting in a decrease in the number of strongly bound cross‐bridges at the fibre level (Caremani et al., 2008; Kawai & Halvorson, 1991). However, cross‐bridge detachment (2π*c*) was slower in MHC I fibres or unchanged in MHC IIA fibres in humans at 37°C (Figure 6b, left panel). Our findings that low pH did not lower force could be attributable to the rate of cross‐bridge attachment being more sensitive to increased temperature than the rate of cross‐bridge detachment (Debold et al., 2004; Momb et al., 2025; Wang & Kawai, 2001). Thereby, the purported mechanism of decreased force with pH, slowing of the forward rate constant (Jarvis et al., 2018; Metzger & Moss, 1990; Woodward & Debold, 2018), might be mitigated at higher temperatures.

### 4.5 Effects of P_i_ or pH on cross‐bridge kinetics

Previous studies have also found that cross‐bridge kinetics respond to P_i_ or pH alone in a fibre type‐specific manner (Millar & Homsher, 1992; Potma et al., 1995; Tesi et al., 2000, 2002; Wahr et al., 1997). Increasing P_i_ alone in slow‐contracting fibres or myofibrils caused *k*_tr_ to be unchanged over the range 0–20 mM (Tesi et al., 2000, 2002; Wahr et al., 1997), but decreased at 30 mM (Wahr et al., 1997). Increasing P_i_ alone in fast‐contracting fibres caused a very different response, with *k*_tr_ increasing at lower concentrations (Tesi et al., 2000, 2002; Wahr et al., 1997), then not changing at concentrations of >10 mM (Wahr et al., 1997). The exponential rate of isometric force decline when caged P_i_ is released by laser flash photolysis, or *k*_Pi_, represents the effect of P_i_ binding on strongly bound cross‐bridges (Dantzig et al., 1992). Slow‐contracting fibres showed similar rates for *k*_Pi_ and *k*_tr_, whereas fast‐contracting fibres showed higher rates for *k*_Pi_ compared with *k*_tr_ (Millar & Homsher, 1992), providing further evidence that P_i_ alone alters cross‐bridge kinetics in a manner that depends on fibre type. Examining pH alone, the myosin ATPase rate slows in slow‐contracting fibres and quickens in fast‐contracting fibres contracting isometrically (Potma et al., 1995). Overall, these studies indicate that increased P_i_ or decreased pH alone show that cross‐bridge kinetics decrease in slow‐contracting fibres and increase or remain unchanged in fast‐contracting fibres, in agreement with our present work at 37°C.

### 4.6 Effects of P_i_ and/or pH on oscillatory work

To understand how changes to the underlying cross‐bridge mechanics and kinetics combine to alter contractile function, we analysed the performance of molecular motors by measuring oscillatory work at 37°C. When manipulating P_i_ or pH independently in MHC I and IIA fibres, reduced or negative work occurred (Figure 7), with no change in specific tension (Figure 2). This was a result of reduced *B* and *C* in MHC I fibres, but an increased *C*/*B* ratio in MHC IIA fibres (Figures 4 and 5). Although not a direct comparison, depression of oscillatory work occurs without reduced force production in *Drosophila* with mutations to flightin (Henkin et al., 2004), showing that performance of molecular motors can change without alterations to force. Our findings indicate that the ability to generate work might be compromised when altering P_i_ or pH alone, even if cellular level force is not changing substantially. Notably, oscillatory work during fatiguing conditions in MHC I and IIA fibres was similar or closer to control conditions, indicating an interacting effect of these metabolites. In MHC I fibres, this occurs because as the oscillatory frequency of the C‐process increases, the amount of work absorption at the lower oscillatory frequencies (where positive work output occurs) decreases. Thus, altering 2π*c*/2π*b* with fatigue in MHC I fibres causes the fatigue response in these fibres to more closely resemble control than P_i_ or pH alone. In MHC IIA fibres, by modelling the transition of 2π*b* and 2π*c*, *B* and *C*, or a combination of all four back to control levels, we found the greatest impact to work production via the combination of all four (Figure 7d). Although *B*, *C* and their ratio were not statistically different (Figure 5), the subtle change in *C*/*B* from 107% in fatigue to 105% in control conditions created a shift from work absorption (process C) to work production (process B). Thus, although a primary difference between fatigue and control is an increase in myofilament viscosity in MHC IIA fibres, peak work with fatigue is reduced primarily owing to alterations in work production and work absorption. These findings further quantify the interacting effects of P_i_ and pH at 37°C, with fibre type specificity. Importantly, MHC I fibres displayed a general fatigue resistance regarding oscillatory work when high P_i_ and low pH were combined, highlighting important fibre type‐specific differences in in vivo metabolite accumulation.

### 4.7 Limitations

There are several important issues to remember when evaluating our work. First, multiple other factors contribute to skeletal muscle fatigue in vivo, such as impaired calcium release from the sarcoplasmic reticulum, reduced ATP or increased Mg^2+^, glycogen depletion, impaired excitability of the sarcolemma, changes to reactive oxygen and/or nitrogen species, in addition to reduced calcium sensitivity of single fibres (Debold & Westerblad, 2024). Thus, the present work should remain within the context of increased P_i_ and reduced pH and not the entire milieu associated with muscle fatigue. Second, our observations are made in isometric conditions at a specific sarcomere length (2.65 µm), which might produce different cross‐bridge kinetics in comparison to shorter or longer sarcomere lengths (Fenwick et al., 2021) and when fibres are shortening (Nyitrai & Geeves, 2004), hence we cautiously extrapolate our present findings for isometric single fibres to other conditions. Third, we acknowledge that this work was performed in fibres from older adults, which might respond differently to fatigue compared with fibres from young adults, especially given that our techniques have shown that ageing alters both force production and cross‐bridge kinetics (Miller et al., 2013).

## 5 CONCLUSION

Our data bring to light new evidence of the effects of fatigue on human skeletal muscle single‐fibre contractile function at a physiological temperature (37°C). Specifically, molecular‐level contractile function is different between slow‐ and fast‐contracting fibres when altering P_i_ or pH independently or in combination. The fatigue properties of MHC I and IIA fibres is a crucial area to study, because these MHC isoforms make up the largest proportion of human skeletal muscle, compared with relatively small amounts of hybrid muscle fibres and almost no pure MHC IIX fibres (Murach et al., 2019). Thus, the proportion of each in an individual might influence overall muscle performance during fatigue (Bagley et al., 2017; Thorstensson & Karlsson, 1976). Our findings highlight that although both fibre types exhibit reductions in force production with fatiguing conditions, MHC I fibres might be fatigue resistant during oscillatory work production, whereas MHC IIA fibres are not. Because the mechanisms underlying the response to fatiguing conditions are fibre type specific at 37°C, this indicates a need to examine force–velocity–power profiles at physiological temperatures. Although reductions in peak power occur in both fibre types by 40%–55% in human single fibres (Sundberg et al., 2018) and by ∼60% in rat single fibres (Nelson et al., 2014), these studies were conducted between 15°C and 30°C, and our results suggest that these findings might not translate to in vivo temperature in humans. If feasible, future work should aim to test single‐fibre function, or other molecular preparations, close to or at physiological temperatures for comparison with parallel studies in vivo.

## AUTHOR CONTRIBUTIONS

Brent A. Momb and Mark S. Miller conceived and designed the research. Brent A. Momb and Stuart R. Chipkin acquired human skeletal muscle tissue. Brent A. Momb performed experiments and analysed data. Brent A. Momb, Jane A. Kent, Stuart R. Chipkin and Mark S. Miller interpreted results of research. Brent A. Momb, Jane A. Kent and Mark S. Miller prepared figures. Brent A. Momb and Mark S. Miller drafted the manuscript. All authors contributed to the critical revision of the manuscript, approved the final version of the manuscript and agree to be accountable for all aspects of the work in ensuring that questions related to the accuracy or integrity of any part of the work are appropriately investigated and resolved. All persons designated as authors qualify for authorship, and all those who qualify for authorship are listed.

## CONFLICT OF INTEREST

None.

**AUTHOR'S TRANSLATIONAL PERSPECTIVE**

This work describes and highlights the importance of conducting muscle single‐fibre experiments at or near in vivo conditions. We are interested in understanding how cellular and molecular function of skeletal muscle dictates whole‐muscle and physical function. Our results show that slow‐ and fast‐contracting fibres respond differently to alterations in P_i_ and pH at human body temperature, which should be considered when translating findings from studies using temperatures that are well below physiological to whole‐muscle function. Application of the present approach will allow future work to be more targeted towards understanding human health and disease.

## Data Availability

All data are represented within this manuscript.

## References

[eph70323-bib-0001] Bagley, J. R. , McLeland, K. A. , Arevalo, J. A. , Brown, L. E. , Coburn, J. W. , & Galpin, A. J. (2017). Skeletal muscle fatigability and myosin heavy chain fiber type in resistance trained men. Journal of Strength and Conditioning Research, 31(3), 602–607. 27984439 10.1519/JSC.0000000000001759

[eph70323-bib-0002] Bergh, U. , & Ekblom, B. (1979). Influence of muscle temperature on maximal muscle strength and power output in human skeletal muscles. Acta Physiologica Scandinavica, 107(1), 33–37. 525366 10.1111/j.1748-1716.1979.tb06439.x

[eph70323-bib-0004] Brenner, B. (1988). Effect of Ca^2+^ on cross‐bridge turnover kinetics in skinned single rabbit psoas fibers: Implications for regulation of muscle contraction. Proceedings of the National Academy of Sciences, 85(9), 3265–3269. 10.1073/pnas.85.9.3265 PMC280185 2966401

[eph70323-bib-0005] Brenner, B. (2006). The stroke size of myosins: A reevaluation. Journal of Muscle Research and Cell Motility, 27(2), 173–187. 16470332 10.1007/s10974-006-9056-7

[eph70323-bib-0006] Brenner, B. , Hahn, N. , Hanke, E. , Matinmehr, F. , Scholz, T. , Steffen, W. , & Kraft, T. (2012). Mechanical and kinetic properties of β‐cardiac/slow skeletal muscle myosin. Journal of Muscle Research and Cell Motility, 33(6), 403–417. 22847802 10.1007/s10974-012-9315-8

[eph70323-bib-0007] Broxterman, R. M. , Layec, G. , Hureau, T. J. , Amann, M. , & Richardson, R. S. (2017). Skeletal muscle bioenergetics during all‐out exercise: Mechanistic insight into the oxygen uptake slow component and neuromuscular fatigue. Journal of Applied Physiology, 122(5), 1208–1217. 28209743 10.1152/japplphysiol.01093.2016 PMC5451539

[eph70323-bib-0008] Cady, E. B. , Jones, D. A. , Lynn, J. , & Newham, D. J. (1989). Changes in force and intracellular metabolites during fatigue of human skeletal muscle. The Journal of Physiology, 418(1), 311–325. 2621621 10.1113/jphysiol.1989.sp017842 PMC1189973

[eph70323-bib-0009] Capitanio, M. , Canepari, M. , Cacciafesta, P. , Lombardi, V. , Cicchi, R. , Maffei, M. , Pavone, F. S. , & Bottinelli, R. (2006). Two independent mechanical events in the interaction cycle of skeletal muscle myosin with actin. Proceedings of the National Academy of Sciences, 103(1), 87–92. 10.1073/pnas.0506830102 PMC1324983 16371472

[eph70323-bib-0010] Caremani, M. , Dantzig, J. , Goldman, Y. E. , Lombardi, V. , & Linari, M. (2008). Effect of inorganic phosphate on the force and number of myosin cross‐bridges during the isometric contraction of permeabilized muscle fibers from rabbit psoas. Biophysical Journal, 95(12), 5798–5808. 18835889 10.1529/biophysj.108.130435 PMC2599836

[eph70323-bib-0011] Chen, X. , Sanchez, G. N. , Schnitzer, M. J. , & Delp, S. L. (2016). Changes in sarcomere lengths of the human vastus lateralis muscle with knee flexion measured using in vivo microendoscopy. Journal of Biomechanics, 49(13), 2989–2994. 27481293 10.1016/j.jbiomech.2016.07.013 PMC5507365

[eph70323-bib-0012] Cooke, R. (2007). Modulation of the actomyosin interaction during fatigue of skeletal muscle. Muscle & Nerve, 36(6), 756–777. 17823954 10.1002/mus.20891

[eph70323-bib-0013] Cooke, R. , & Franks, K. (1980). All myosin heads form bonds with actin in rigor rabbit skeletal muscle. Biochemistry, 19(10), 2265–2269. 6103713 10.1021/bi00551a042

[eph70323-bib-0014] Cooke, R. , Franks, K. , Luciani, G. , & Pate, E. (1988). The inhibition of rabbit skeletal muscle contraction by hydrogen ions and phosphate. The Journal of Physiology, 395(1), 77–97. 2842489 10.1113/jphysiol.1988.sp016909 PMC1191984

[eph70323-bib-0015] Cooke, R. , & Pate, E. (1985). The effects of ADP and phosphate on the contraction of muscle fibers. Biophysical Journal, 48(5), 789–798. 3878160 10.1016/S0006-3495(85)83837-6 PMC1329404

[eph70323-bib-0016] Coupland, M. E. , Puchert, E. , & Ranatunga, K. W. (2001). Temperature dependance of active tension in mammalian (rabbit psoas) muscle fibres: Effect of inorganic phosphate. The Journal of Physiology, 536(Pt 3), 879–891. 11691880 10.1111/j.1469-7793.2001.00879.x PMC2278902

[eph70323-bib-0017] Dantzig, J. A. , Goldman, Y. E. , Millar, N. C. , Lacktis, J. , & Homsher, E. (1992). Reversal of the cross‐bridge force‐generating transition by photogeneration of phosphate in rabbit psoas muscle fibres. The Journal of Physiology, 451(1), 247–278. 1403812 10.1113/jphysiol.1992.sp019163 PMC1176160

[eph70323-bib-0018] Debold, E. P. , Beck, S. E. , & Warshaw, D. M (2008). Effect of low pH on single skeletal muscle myosin mechanics and kinetics. American Journal of Physiology‐Cell Physiology, 295(1), C173–C179. 18480297 10.1152/ajpcell.00172.2008 PMC2493560

[eph70323-bib-0019] Debold, E. P. , Dave, H. , & Fitts, R. H (2004). Fiber type and temperature dependence of inorganic phosphate: Implications for fatigue. American Journal of Physiology‐Cell Physiology, 287(3), C673–C681. 15128502 10.1152/ajpcell.00044.2004

[eph70323-bib-0020] Debold, E. P. , & Westerblad, H. (2024). New insights into the cellular and molecular mechanisms of skeletal muscle fatigue: The Marion J. Siegman Award Lectureships. American Journal of Physiology‐Cell Physiology, 327(4), C946–C958. 39069825 10.1152/ajpcell.00213.2024

[eph70323-bib-0021] Fenwick, A. J. , Lin, D. C. , & Tanner, B. C. W. (2021). Myosin cross‐bridge kinetics slow at longer muscle lengths during isometric contractions in intact soleus from mice. Proceedings of the Royal Society B: Biological Sciences, 288(1950), 20202895. 10.1098/rspb.2020.2895 PMC8190544 33975478

[eph70323-bib-0022] Fitzgerald, L. F. , Bartlett, M. F. , & Kent, J. A. (2023). Muscle fatigue, bioenergetic responses and metabolic economy during load‐ and velocity‐based maximal dynamic contractions in young and older adults. Physiological Reports, 11(22), e15876. 37996974 10.14814/phy2.15876 PMC10667588

[eph70323-bib-0023] Flouris, A. D. , Webb, P. , & Kenny, G. P. (2015). Noninvasive assessment of muscle temperature during rest, exercise, and postexercise recovery in different environments. Journal of Applied Physiology, 118(10), 1310–1320. 25814638 10.1152/japplphysiol.00932.2014 PMC4436983

[eph70323-bib-0024] Foster, A. D. , Straight, C. R. , Woods, P. C. , Lee, C. , Kent, J. A. , Chipkin, S. R. , Debold, E. P. , & Miller, M. S. (2025). Cellular and molecular contractile function in aged human skeletal muscle is altered by phosphate and acidosis and partially reversed with an ATP analog. American Journal of Physiology‐Cell Physiology, 328(4), C1220–C1233. 40047118 10.1152/ajpcell.00332.2024 PMC12225719

[eph70323-bib-0026] Godt, R. E. , & Lindley, B. D. (1982). Influence of temperature upon contractile activation and isometric force production in mechanically skinned muscle fibers of the frog. The Journal of General Physiology, 80(2), 279–297. 6981684 10.1085/jgp.80.2.279 PMC2228673

[eph70323-bib-0027] Henkin, J. A. , Maughan, D. W. , & Vigoreaux, J. O. (2004). Mutations that affect flightin expression in drosophila alter the viscoelastic properties of flight muscle fibers. American Journal of Physiology‐Cell Physiology, 286(1), C65–C72. 12954604 10.1152/ajpcell.00257.2003

[eph70323-bib-0028] Jarvis, K. , Woodward, M. , Debold, E. P. , & Walcott, S. (2018). Acidosis affects muscle contraction by slowing the rates myosin attaches to and detaches from actin. Journal of Muscle Research and Cell Motility, 39(3–4), 135–147. 30382520 10.1007/s10974-018-9499-7

[eph70323-bib-0029] Karatzaferi, C. , Franks‐Skiba, K. , & Cooke, R. (2008). Inhibition of shortening velocity of skinned skeletal muscle fibers in conditions that mimic fatigue. American Journal of Physiology‐Regulatory, Integrative and Comparative Physiology, 294(3), R948–R955. 18077511 10.1152/ajpregu.00541.2007

[eph70323-bib-0030] Kawai, M. (2003). What do we learn by studying the temperature effect on isometric tension and tension transients in mammalian striated muscle fibres? Journal of Muscle Research & Cell Motility, 24(2‐3), 127–138. 14609024 10.1023/a:1026093212111

[eph70323-bib-0032] Kawai, M. , & Halvorson, H. R. (1991). Two step mechanism of phosphate release and the mechanism of force generation in chemically skinned fibers of rabbit psoas muscle. Biophysical Journal, 59(2), 329–342. 2009356 10.1016/S0006-3495(91)82227-5 PMC1281150

[eph70323-bib-0033] Kawai, M. , Saeki, Y. , & Zhao, Y. (1993). Crossbridge scheme and the kinetic constants of elementary steps deduced from chemically skinned papillary and trabecular muscles of the ferret. Circulation Research, 73(1), 35–50. 8508533 10.1161/01.res.73.1.35

[eph70323-bib-0034] Kemp, G. J. , Meyerspeer, M. , & Moser, E. (2007). Absolute quantification of phosphorus metabolite concentrations in human muscle in vivo by 31P MRS: A quantitative review. NMR in Biomedicine, 20(6), 555–565. 17628042 10.1002/nbm.1192

[eph70323-bib-0035] Kenny, G. P. , Reardon, F. D. , Zaleski, W. , Reardon, M. L. , Haman, F. , & Ducharme, M. B. (2003). Muscle temperature transients before, during, and after exercise measured using an intramuscular multisensor probe. Journal of Applied Physiology, 94(6), 2350–2357. 12598487 10.1152/japplphysiol.01107.2002

[eph70323-bib-0036] Kent‐Braun, J. A. (1999). Central and peripheral contributions to muscle fatigue in humans during sustained maximal effort. European Journal of Applied Physiology and Occupational Physiology, 80(1), 57–63. 10367724 10.1007/s004210050558

[eph70323-bib-0037] Kent‐Braun, J. A. , Miller, R. G. , & Weiner, M. W. (1993). Phases of metabolism during progressive exercise to fatigue in human skeletal muscle. Journal of Applied Physiology, 75(2), 573–580. 8226454 10.1152/jappl.1993.75.2.573

[eph70323-bib-0038] Kent‐Braun, J. A. , Ng, A. V. , Doyle, J. W. , & Towse, T. F. (2002). Human skeletal muscle responses vary with age and gender during fatigue due to incremental isometric exercise. Journal of Applied Physiology, 93(5), 1813–1823. 12381770 10.1152/japplphysiol.00091.2002

[eph70323-bib-0039] Knuth, S. T. , Dave, H. , Peters, J. R. , & Fitts, R. H. (2006). Low cell pH depresses peak power in rat skeletal muscle fibres at both 30°C and 15°C: Implications for muscle fatigue. The Journal of Physiology, 575(Pt 3), 887–899. 16809373 10.1113/jphysiol.2006.106732 PMC1995695

[eph70323-bib-0040] Linari, M. , Bottinelli, R. , Pellegrino, M. A. , Reconditi, M. , Reggiani, C. , & Lombardi, V. (2004). The mechanism of the force response to stretch in human skinned muscle fibres with different myosin isoforms. The Journal of Physiology, 554(Pt 2), 335–352. 14555725 10.1113/jphysiol.2003.051748 PMC1664769

[eph70323-bib-0041] Lovell, S. J. , Knight, P. J. , & Harrington, W. F. (1981). Fraction of myosin heads bound to thin filaments in rigor fibrils from insect flight and vertebrate muscles. Nature, 293(5834), 664–666. 7290203 10.1038/293664a0

[eph70323-bib-0042] Marang, C. P. , Petersen, D. J. , Scott, B. D. , Walcott, S. , & Debold, E. P. (2025). Characterizing the concentration and load dependence of phosphate binding to rabbit fast skeletal actomyosin. Proceedings of the National Academy of Sciences, 122(20), e2504758122. 10.1073/pnas.2504758122 PMC12107134 40359046

[eph70323-bib-0043] Metzger, J. M. , & Moss, R. L. (1987). Greater hydrogen ion‐induced depression of tension and velocity in skinned single fibres of rat fast than slow muscles. The Journal of Physiology, 393(1), 727–742. 3446809 10.1113/jphysiol.1987.sp016850 PMC1192420

[eph70323-bib-0044] Metzger, J. M. , & Moss, R. L. (1990). pH modulation of the kinetics of a Ca^2^ ^+^‐sensitive cross‐bridge state transition in mammalian single skeletal muscle fibres. The Journal of Physiology, 428(1), 751–764. 2231432 10.1113/jphysiol.1990.sp018239 PMC1181674

[eph70323-bib-0045] Millar, N. C. , & Homsher, E. (1992). Kinetics of force generation and phosphate release in skinned rabbit soleus muscle fibers. American Journal of Physiology‐Cell Physiology, 262(5 Pt 1), C1239–C1245. 10.1152/ajpcell.1992.262.5.C1239 1590362

[eph70323-bib-0046] Miller, M. S. , Bedrin, N. G. , Callahan, D. M. , Previs, M. J. , Jennings, M. E. , Ades, P. A. , Maughan, D. W. , Palmer, B. M. , & Toth, M. J. (2013). Age‐related slowing of myosin actin cross‐bridge kinetics is sex specific and predicts decrements in whole skeletal muscle performance in humans. Journal of Applied Physiology, 115(7), 1004–1014. 23887900 10.1152/japplphysiol.00563.2013 PMC3798822

[eph70323-bib-0047] Miller, M. S. , & Toth, M. J. (2013). Myofilament protein alterations promote physical disability in aging and disease. Exercise and Sport Sciences Reviews, 41(2), 93–99. 23392279 10.1097/JES.0b013e31828bbcd8 PMC4171103

[eph70323-bib-0048] Miller, M. S. , VanBuren, P. , LeWinter, M. M. , Braddock, J. M. , Ades, P. A. , Maughan, D. W. , Palmer, B. M. , & Toth, M. J. (2010). Chronic heart failure decreases cross‐bridge kinetics in single skeletal muscle fibres from humans. The Journal of Physiology, 588(Pt 20), 4039–4053. 20724360 10.1113/jphysiol.2010.191957 PMC3000591

[eph70323-bib-0049] Mizuno, M. , Horn, A. , Secher, N. H. , Mizuno, A. , Horn, N. H. , & Secher, B. Q. (1994a). Exercise‐induced 31P‐NMR metabolic response of human wrist flexor muscles during partial neuromuscular blockade. American Physiological Society, 267(2 Pt 2), 408–414. 10.1152/ajpregu.1994.267.2.R408 7915086

[eph70323-bib-0050] Mizuno, M. , Secher, N. H. , & Quistorff, B. (1994b). 31P‐NMR spectroscopy, rsEMG, and histochemical fiber types of human wrist flexor muscles. Journal of Applied Physiology, 76(2), 531–538. 8175559 10.1152/jappl.1994.76.2.531

[eph70323-bib-0051] Momb, B. A. , Chipkin, S. R. , Kent, J. A. , & Miller, M. S. (2025). Slow‐ and fast‐contracting skeletal muscle fibers have more similar cellular and molecular contractile function at 37°C than at 25°C in older adults. *bioRxiv*. 10.1101/2025.08.27.670366 PMC13423134 42459137

[eph70323-bib-0052] Momb, B. A. , Patino, E. , Akchurin, O. M. , & Miller, M. S. (2022). Iron supplementation improves skeletal muscle contractile properties in mice with chronic kidney disease. Kidney360, 3(5), 843–858. 36128477 10.34067/KID.0004412021 PMC9438424

[eph70323-bib-0053] Mulieri, L. A. , Barnes, W. , Leavitt, B. J. , Ittleman, F. P. , LeWinter, M. M. , Alpert, N. R. , & Maughan, D. W. (2002). Alterations of myocardial dynamic stiffness implicating abnormal crossbridge function in human mitral regurgitation heart failure. Circulation Research, 90(1), 66–72. 11786520 10.1161/hh0102.103221

[eph70323-bib-0054] Murach, K. A. , Dungan, C. M. , Kosmac, K. , Voigt, T. B. , Tourville, T. W. , Miller, M. S. , Bamman, M. M. , Peterson, C. A. , & Toth, M. J. (2019). Fiber typing human skeletal muscle with fluorescent immunohistochemistry. Journal of Applied Physiology, 127(6), 1632–1639. 31697594 10.1152/japplphysiol.00624.2019 PMC6957370

[eph70323-bib-0055] Nelson, C. R. , Debold, E. P. , & Fitts, R. H. (2014). Phosphate and acidosis act synergistically to depress peak power in rat muscle fibers. American Journal of Physiology‐Cell Physiology, 307(10), C939–C950. 25186012 10.1152/ajpcell.00206.2014 PMC4233260

[eph70323-bib-0056] Nocella, M. , Colombini, B. , Benelli, G. , Cecchi, G. , Bagni, M. A. , & Bruton, J. (2011). Force decline during fatigue is due to both a decrease in the force per individual cross‐bridge and the number of cross‐bridges. The Journal of Physiology, 589(Pt 13), 3371–3381. 21540343 10.1113/jphysiol.2011.209874 PMC3145945

[eph70323-bib-0057] Nyitrai, M. , & Geeves, M. A. (2004). Adenosine diphosphate and strain sensitivity in myosin motors. Philosophical Transactions of the Royal Society of London. Series B: Biological Sciences, 359(1452), 1867–1877. 15647162 10.1098/rstb.2004.1560 PMC1693474

[eph70323-bib-0058] Nyitrai, M. , Rossi, R. , Adamek, N. , Pellegrino, M. A. , Bottinelli, R. , & Geeves, M. A. (2006). What limits the velocity of fast‐skeletal muscle contraction in mammals? Journal of Molecular Biology, 355(3), 432–442. 16325202 10.1016/j.jmb.2005.10.063

[eph70323-bib-0059] Palmer, B. M. , Fishbaugher, D. E. , Schmitt, J. P. , Wang, Y. , Alpert, N. R. , Seidman, C. E. , Seidman, J. G. , VanBuren, P. , & Maughan, D. W. (2004). Differential cross‐bridge kinetics of FHC myosin mutations R403Q and R453C in heterozygous mouse myocardium. American Journal of Physiology‐Heart and Circulatory Physiology, 287(1), H91–H99. 15001446 10.1152/ajpheart.01015.2003

[eph70323-bib-0060] Palmer, B. M. , Suzuki, T. , Wang, Y. , Barnes, W. D. , Miller, M. S. , & Maughan, D. W. (2007). Two‐state model of acto‐myosin attachment‐detachment predicts C‐process of sinusoidal analysis. Biophysical Journal, 93(3), 760–769. 17496022 10.1529/biophysj.106.101626 PMC1913148

[eph70323-bib-0061] Palmer, B. M. , Tanner, B. C. W. , Toth, M. J. , & Miller, M. S. (2013). An inverse power‐law distribution of molecular bond lifetimes predicts fractional derivative viscoelasticity in biological tissue. Biophysical Journal, 104(11), 2540–2552. 23746527 10.1016/j.bpj.2013.04.045 PMC3672888

[eph70323-bib-0062] Pate, E. , Bhimani, M. , Franks‐Skiba, K. , & Cooke, R. (1995). Reduced effect of pH on skinned rabbit psoas muscle mechanics at high temperatures: Implications for fatigue. The Journal of Physiology, 486(Pt 3), 689–694. 7473229 10.1113/jphysiol.1995.sp020844 PMC1156556

[eph70323-bib-0063] Pathare, N. , Walter, G. A. , Stevens, J. E. , Yang, Z. , Okerke, E. , Gibbs, J. D. , Esterhai, J. L. , Scarborough, M. T. , Gibbs, C. P. , Sweeney, H. L. , & Vandenborne, K. (2005). Changes in inorganic phosphate and force production in human skeletal muscle after cast immobilization. Journal of Applied Physiology, 98(1), 307–314. 15333614 10.1152/japplphysiol.00612.2004

[eph70323-bib-0064] Pellegrino, M. A. , Canepari, M. , Rossi, R. , D'Antona, G. , Reggiani, C. , & Bottinelli, R. (2003). Orthologous myosin isoforms and scaling of shortening velocity with body size in mouse, rat, rabbit and human muscles. The Journal of Physiology, 546(Pt 3), 677–689. 12562996 10.1113/jphysiol.2002.027375 PMC2342590

[eph70323-bib-0065] Potma, E. J. , van Graas, I. A. , & Stienen, G. J. (1995). Influence of inorganic phosphate and pH on ATP utilization in fast and slow skeletal muscle fibers. Biophysical Journal, 69(6), 2580–2589. 8599665 10.1016/S0006-3495(95)80129-3 PMC1236496

[eph70323-bib-0067] Sundberg, C. W. , Hunter, S. K. , Trappe, S. W. , Smith, C. S. , & Fitts, R. H. (2018). Effects of elevated H^+^ and P_i_ on the contractile mechanics of skeletal muscle fibres from young and old men: Implications for muscle fatigue in humans. The Journal of Physiology, 596(17), 3993–4015. 29806714 10.1113/JP276018 PMC6117549

[eph70323-bib-0068] Sundberg, C. W. , Teigen, L. E. , Hunter, S. K. , & Fitts, R. H. (2025). Cumulative effects of H^+^ and P_i_ on force and power of skeletal muscle fibres from young and older adults. The Journal of Physiology, 603(1), 187–209. 39545875 10.1113/JP286938 PMC11702922

[eph70323-bib-0069] Tesi, C. , Colomo, F. , Nencini, S. , Piroddi, N. , & Poggesi, C. (2000). The effect of inorganic phosphate on force generation in single myofibrils from rabbit skeletal muscle. Biophysical Journal, 78(6), 3081–3092. 10827985 10.1016/S0006-3495(00)76845-7 PMC1300890

[eph70323-bib-0070] Tesi, C. , Colomo, F. , Piroddi, N. , & Poggesi, C. (2002). Characterization of the cross‐bridge force‐generating step using inorganic phosphate and BDM in myofibrils from rabbit skeletal muscles. The Journal of Physiology, 541(Pt 1), 187–199. 12015429 10.1113/jphysiol.2001.013418 PMC2315793

[eph70323-bib-0071] Thorstensson, A. , & Karlsson, J. (1976). Fatiguability and fibre composition of human skeletal muscle. Acta Physiologica Scandinavica, 98(3), 318–322. 136865 10.1111/j.1748-1716.1976.tb10316.x

[eph70323-bib-0072] Troiano, R. P. , Berrigan, D. , Dodd, K. W. , Mâsse, L. C. , Tilert, T. , & Mcdowell, M. (2008). Physical activity in the United States measured by accelerometer. Medicine & Science in Sports & Exercise, 40(1), 181–188. 18091006 10.1249/mss.0b013e31815a51b3

[eph70323-bib-0073] Tyska, M. J. , & Warshaw, D. M. (2002). The myosin power stroke. Cell Motility, 51(1), 1–15. 10.1002/cm.10014 11810692

[eph70323-bib-0074] Vandenboom, R. (2017). Modulation of skeletal muscle contraction by myosin phosphorylation. Comprehensive Physiology, 7(1), 171–212. 10.1002/cphy.c150044 28135003

[eph70323-bib-0075] Wahr, P. A. , Cantor, H. C. , & Metzger, J. M. (1997). Nucleotide‐dependent contractile properties of Ca2^+^‐activated fast and slow skeletal muscle fibers. Biophysical Journal, 72(2 Pt 1), 822–834. 9017207 10.1016/s0006-3495(97)78716-2 PMC1185605

[eph70323-bib-0076] Wang, G. , & Kawai, M. (2001). Effect of temperature on elementary steps of the cross‐bridge cycle in rabbit soleus slow‐twitch muscle fibres. The Journal of Physiology, 531(Pt 1), 219–234. 11179405 10.1111/j.1469-7793.2001.0219j.x PMC2278446

[eph70323-bib-0077] Wang, Y. , Tanner, B. C. W. , Lombardo, A. T. , Tremble, S. M. , Maughan, D. W. , VanBuren, P. , LeWinter, M. M. , Robbins, J. , & Palmer, B. M. (2013). Cardiac myosin isoforms exhibit differential rates of MgADP release and MgATP binding detected by myocardial viscoelasticity. Journal of Molecular and Cellular Cardiology, 54, 1–8. 23123290 10.1016/j.yjmcc.2012.10.010 PMC3535576

[eph70323-bib-0078] Weiner, M. W. , Moussavi, R. S. , Baker, A. J. , Boska, M. D. , & Miller, R. G. (1990). Constant relationships between force, phosphate concentration, and pH in muscles with differential fatigability. Neurology, 40(12), 1888–1893. 2247239 10.1212/wnl.40.12.1888

[eph70323-bib-0079] Westerblad, H. , Bruton, J. D. , & Lännergren, J. (1997). The effect of intracellular pH on contractile function of intact, single fibres of mouse muscle declines with increasing temperature. The Journal of Physiology, 500(Pt 1), 193–204. 9097943 10.1113/jphysiol.1997.sp022009 PMC1159369

[eph70323-bib-0080] Widrick, J. J. (2002). Effect of Pi on unloaded shortening velocity of slow and fast mammalian muscle fibers. American Journal of Physiology‐Cell Physiology, 282(4), C647–C653. 11880253 10.1152/ajpcell.00186.2001

[eph70323-bib-0081] Wiseman, R. W. , Beck, T. W. , & Chase, P. B. (1996). Effect of intracellular pH on force development depends on temperature in intact skeletal muscle from mouse. American Journal of Physiology‐Cell Physiology, 271(3 Pt 1), C878–C886. 10.1152/ajpcell.1996.271.3.C878 8843718

[eph70323-bib-0082] Woods, P. C. , Swank, D. M. , & Miller, M. S. (2025). Stretch activation combats force loss from fatigue in fast‐contracting mouse skeletal muscle fibers. Journal of General Physiology, 157(5), e202413679. 40788262 10.1085/jgp.202413679 PMC12406957

[eph70323-bib-0083] Woodward, M. , & Debold, E. P. (2018). Acidosis and phosphate directly reduce myosin's force‐generating capacity through distinct molecular mechanisms. Frontiers in Physiology, 9, 862. 30042692 10.3389/fphys.2018.00862 PMC6048269

[eph70323-bib-0084] Zhao, Y. , & Kawai, M. (1993). The effect of the lattice spacing change on cross‐bridge kinetics in chemically skinned rabbit psoas muscle fibers. II. Elementary steps affected by the spacing change. Biophysical Journal, 64(1), 197–210. 7679297 10.1016/S0006-3495(93)81357-2 PMC1262317

[eph70323-bib-0085] Zhao, Y. , & Kawai, M. (1994). Kinetic and thermodynamic studies of the cross‐bridge cycle in rabbit psoas muscle fibers. Biophysical Journal, 67(4), 1655–1668. 7819497 10.1016/S0006-3495(94)80638-1 PMC1225527

